# Evolutionarily conserved morphogenetic movements at the vertebrate head–trunk interface coordinate the transport and assembly of hypopharyngeal structures

**DOI:** 10.1016/j.ydbio.2014.03.003

**Published:** 2014-06-15

**Authors:** Corinne Lours-Calet, Lucia E. Alvares, Amira S. El-Hanfy, Saniel Gandesha, Esther H. Walters, Débora Rodrigues Sobreira, Karl R. Wotton, Erika C. Jorge, Jennifer A. Lawson, A. Kelsey Lewis, Masazumi Tada, Colin Sharpe, Gabrielle Kardon, Susanne Dietrich

**Affiliations:** a School of Biomedical & Health Sciences, King׳s College London, Hodgkin Building G43S/44S, Guy׳s Campus, London SE1 1UL, UK; b GReD – Génétique Reproduction et Développement, UMR CNRS 6247, INSERM U931, Clermont Université, 24, Avenue des Landais, BP 80026, 63171 Aubiere Cedex, France; c Department of Histology and Embryology, University of Campinas (UNICAMP), Rua Charles Darwin s/n, Cx. Postal 6109, CEP 13083-863 Campinas, São Paulo, Brazil; d College Road Dental Practice, 2 College Road, Bromsgrove, B60 2NE; e Institute for Biomedical and Biomolecular Science (IBBS), School of Pharmacy and Biomedical Sciences, University of Portsmouth, St. Michael׳s Building, White Swan Road, Portsmouth PO1 2DT, UK; f EMBL/CRG Systems Biology Research Unit, Centre for Genomic Regulation (CRG) and UPF, Dr. Aiguader 88, 08003 Barcelona, Spain; g Departamento de Morfologia, Instituto de Ciências Biológicas, Universidade Federal de Minas Gerais (UFMG), Belo Horizonte, Minas Gerais, Brazil; h Department of Human Genetics, University of Utah, 15 North 2030 East, Salt Lake City, UT 84112, USA; i Department of Cell & Developmental Biology, University College London, Gower Street, London WC1E 6BT, UK; j Institute for Biomedical and Biomolecular Science (IBBS), School of Biology, University of Portsmouth, St. Michael׳s Building, White Swan Road, Portsmouth PO1 2DT, UK

**Keywords:** Evolution of vertebrate developmental mechanisms, Head–trunk interface, Morphogenetic movements, Occipital lateral mesoderm, Occipital somites, Occipital ectoderm, Occipital neural crest, Hypobranchial/hypoglossal muscle, Migratory muscle precursors, Floor of pharynx, Pharyngeal arches, Circumpharyngeal route, Zebrafish, *Xenopus*, Chicken, Mouse

## Abstract

The vertebrate head–trunk interface (occipital region) has been heavily remodelled during evolution, and its development is still poorly understood. In extant jawed vertebrates, this region provides muscle precursors for the throat and tongue (hypopharyngeal/hypobranchial/hypoglossal muscle precursors, HMP) that take a stereotype path rostrally along the pharynx and are thought to reach their target sites via active migration. Yet, this projection pattern emerged in jawless vertebrates before the evolution of migratory muscle precursors. This suggests that a so far elusive, more basic transport mechanism must have existed and may still be traceable today.

Here we show for the first time that all occipital tissues participate in well-conserved cell movements. These cell movements are spearheaded by the occipital lateral mesoderm and ectoderm that split into two streams. The rostrally directed stream projects along the floor of the pharynx and reaches as far rostrally as the floor of the mandibular arch and outflow tract of the heart. Notably, this stream leads and engulfs the later emerging HMP, neural crest cells and hypoglossal nerve. When we (i) attempted to redirect hypobranchial/hypoglossal muscle precursors towards various attractants, (ii) placed non-migratory muscle precursors into the occipital environment or (iii) molecularly or (iv) genetically rendered muscle precursors non-migratory, they still followed the trajectory set by the occipital lateral mesoderm and ectoderm. Thus, we have discovered evolutionarily conserved morphogenetic movements, driven by the occipital lateral mesoderm and ectoderm, that ensure cell transport and organ assembly at the head–trunk interface.

## Highlights

• At the vertebrate head–trunk interface, all tissues engage in stereotype cell movements.
• A ventrally–rostrally directed stream of cells leads along the floor of the pharynx to the developing jaw and outflow tract of the heart.
• The cell movements are spearheaded by the lateral mesoderm and surface ectoderm; muscle precursors for throat and tongue muscles (hypopharyngeal muscles); neural crest cells and outgrowing axons of the hypoglossal nerve follow.
• Hypopharyngeal muscle precursors follow the trajectory set by the lateral mesoderm and ectoderm, even when challenged with ectopic attractants or when rendered non-migratory.
• The newly discovered cell movements are the likely ground state for cell transport and organ assembly at the head–trunk interface before actively migrating muscle precursors evolved in “bony” (osteichthyan) vertebrates.

## Introduction

During the evolution of vertebrates, the ancestral lay-out of the pharynx was remodelled, adapting from filter feeding to respiration and active predation (reviewed in (Ericsson et al., 2013; Goodrich, 1958)). Key steps in this process were the deployment of skeletal elements and associated muscle inside the segmented pharyngeal arches, in jawed vertebrates followed by the evolution of jaws from the first pharyngeal arch. A further important step was the evolution of a hypopharyngeal/hypobranchial/hypoglossal support system with muscles oriented longitudinally along the floor of the pharynx that assist the depression of pharyngeal arches during food acquisition and swallowing. In jawed vertebrates, the hypobranchial muscles, via depression of the hyoid bone, facilitate jaw opening; in tetrapods these muscles extend far rostrally into the floor of the mandibular arch, forming the muscles of the tongue that are essential for food uptake on land, mastication, grooming and in humans, speech.

Anatomical and fate mapping studies in numerous species showed that the hypopharyngeal musculature is derived from the occipital somites (Bladt et al., 1995; Couly et al., 1993; Diogo et al., 2008; Edgeworth, 1907; Huang et al., 2000; Kallius, 1901; Kuratani et al., 1999; Kusakabe et al., 2011; Martin and Harland, 2006; Neal, 1897; Noden, 1983; Wachtler and Jacob, 1986); reviewed in (Edgeworth, 1935; Ericsson et al., 2013; Goodrich, 1958; Noden and Francis-West, 2006). Somites are segmental units of paraxial mesoderm that initially belonged to the trunk, supplying muscle for locomotion and later, the vertebral column and ribs (reviewed in (Gilbert, 2000)). During vertebrate evolution, however, the rostral-most trunk was incorporated into the head (Gans and Northcutt, 1983), and to date, the occipital region, besides the hypopharyngeal musculature, also provides fused vertebrae that reinforce the skull vault, muscle that links the skull to the body, ectomesenchyme and muscle for the caudal pharyngeal arches, neural crest cells for the enteric nervous system and the heart, and the hypoglossal nerve that innervates most of the hypopharyngeal/hypoglossal musculature (reviewed in (Ericsson et al., 2013)). Concomitant with the incorporation of the occipital region into the head, developmental programs changed. Hypopharyngeal muscle precursors (HMP) for example partially adopted the head programme in that they assemble with neural crest derived rather than mesoderm-derived connective tissue, and that they express markers found in genuine, non-somitic head muscles (Couly et al., 1993; Noden, 1983; Dietrich, unpublished observations). Thus, the occipital region has evolved into a specialised region, so much so that the development of occipital derivatives is still largely obscure.

The hypopharyngeal muscle precursors (HMP), together with the enteric and cardiac crest and the hypoglossal nerve take a well-defined, circumpharyngeal route along the caudal border of the pharyngeal arches and then rostrally along the floor of the arches, reaching territories far rostral to their site of origin ((Kuratani and Kirby, 1992), reviewed by Ericsson et al. (2013), Goodrich (1958), and Kuo and Erickson (2011); this study). Neural crest cells are known to actively migrate, axons actively search for their targets, and at least in osteichthyan vertebrates (commonly referred to as “bony” vertebrates, but recent fossil findings suggest that relatives of today׳s cartilaginous vertebrates were also able to generate bone; (Zhu et al., 2013)), HMP are thought to actively migrate to their target site as they express many genes associated with the formation of migratory muscle precursors for the paired fins/limbs (reviewed by Gilbert (2000), Noden and Francis-West (2006), Dietrich et al. (1999, 1998), Kusakabe et al. (2011), Martin and Harland (2006), Neyt et al. (2000), Ochi and Westerfield (2009), Sabo et al. (2010), and Thisse et al. (2004); this study). Thus, it is currently thought that these occipital derivatives all undertake a targeted migration. Yet anatomical studies in agnathans and in cartilaginous gnathostomes suggested that these animals have not yet evolved migratory muscle precursors and that hypopharyngeal and fin muscles are laid down via somitic extensions rather than migratory cells (Cole et al., 2011; Kuratani et al., 1999; Kusakabe et al., 2011; Neal, 1897; Neyt et al., 2000); reviewed in (Edgeworth, 1935; Ericsson et al., 2013; Goodrich, 1958; Noden and Francis-West, 2006). In the mouse representing osteichthyans, mutations of Scatter factor/Hepatocyte growth factor or its receptor cMet prevent deepithelialisation and migration of limb/paired fin muscle precursors but do not prevent the formation of hypopharyngeal muscles (Bladt et al., 1995; Dietrich et al., 1999; Prunotto et al., 2004; this study). In the chicken, also a model for osteichthyans, SF/HGF is not expressed along the circumpharyngeal route (Mackenzie et al., 1998). This suggests that there is a so far elusive, evolutionarily basic and conserved mechanism of cell and tissue transport along the circumpharyngeal route that does not rely on cell migration.

In this study, we mapped cell movements at the head–trunk interface and investigated their significance. We show that in osteichthyans as divergent as zebrafish (ray-finned or actinopterygian vertebrates), *Xenopus*, chicken and mouse (sarcopterygian or lobe-finned/limbed vertebrates), molecular markers for all occipital tissues expand their expression domains along the circumpharyngeal route. Labelling experiments revealed that these changes in gene expression are due to cells undertaking coordinated movements. The lateral mesoderm and overlying ectoderm move first and split into two streams, with the rostral stream anticipating the path of the other hypopharyngeal tissues and reaching as far rostrally as the floor of the mandibular arch and the outflow tract of the heart. As the most rostral occipital lateral mesoderm marks the circumpharyngeal path and embeds the subsequently emerging HMP, we investigated whether its movements may be suited to carry HMP along. To test this we (i) challenged HMP with numerous putative attractants, (ii) grafted non-migratory muscle precursors into the occipital environment or (iii) molecularly or (iv) genetically rendered HMP non-migratory. In all these cases HMP followed their normal path. Thus, we have discovered so far unknown morphogenetic cell movements at the head–trunk interface that we propose are the evolutionarily basic mechanism that coordinates hypopharyngeal cell transport and organ assembly.

## Materials and methods

### Culture and staging of embryos

#### Chicken and quail embryos

Fertilised chicken (Winter Farm, Royston and Henry Stewart Ltd., Norfolk) and Japanese quail eggs (Potter Farm, Woodhurst) were incubated in a humidified atmosphere at 38.5 °C and staged according to (Ainsworth et al., 2010; Hamburger and Hamilton, 1951). Eggs for in ovo manipulations were windowed and non-toxic drawing ink, diluted 1:5 in phosphate buffered saline (PBS), was injected sub-blastodermally. After manipulation, the embryos were moistened with PBS, the eggs sealed with tape and re-incubated at 38.5 °C. Embryos were harvested in 4% PFA.

#### Mouse embryos

Wildtype and Pax3^Spd/+^ mice were mated overnight. The appearance of a vaginal plug the next morning was taken as day 0.5 of development (E0.5). Pregnant females were sacrificed at E10.5 and 14.5 by cervical dislocation and the embryos were fixed in 4% PFA. Homozygous *Sp*^*d*^/*Sp*^*d*^ embryos were identified based on their caudal rachischisis.

#### *Xenopus* embryos

Wildtype and cardiac actin:GFP *Xenopus laevis* embryos were obtained from the European Xenopus Resource Centre (EXRC) at the University of Portsmouth. Embryos were dejellied in 2% cystein–HCl (pH 8.0), grown at 18–23 °C in 0.1× MBS (Gurdon, 1977), staged according to (Nieuwkoop and Faber, 1994) and harvested in MEMFA (Harland, 1991).

#### Zebrafish embryos

Breeding of zebrafish (*Danio rerio*) was maintained at 28 °C on a 14 h light/10 h dark cycle. Embryos were obtained by natural spawning, grown in egg water (0.3 g/l Instant Ocean Salt, 1 mg/l Methylene Blue) at 28 °C and staged according to Kimmel et al. (1995). To prevent pigment formation, embryos post-24 hpf were raised in 0.2 mM 1-phenyl-2-thiourea (PTU, Sigma). Embryos were harvested in 4% PFA.

#### in situ hybridisation, immunohistochemistry, and sectioning

References for protocols, probes and antibodies used for whole mount in situ hybridisation (WISH), double WISH, WISH followed by whole mount antibody staining, vibratome sectioning and antibody staining on cryosections are detailed in Supplementary Material 1.

#### DiI/DiO labelling

0.5% DiI or 0.25% DiO (Invitrogen) in ethanol or in DMSO was applied using an aspirator. Labellings in the chicken were carried out at HH8^+^–HH10^−^, in *Xenopus* at st 20.

#### Molecular constructs

PCR primers were designed that allowed the introduction of an EcoRI site before and an AflII site after the coding region for the chicken Lbx1 EH1 transrepression domain, causing conservative amino acid exchanges only. The restriction sites were used to replace the EH1 domain with the VP16 transactivation domain. The Lbx1-Vp16 open reading frame was cloned into the multiple cloning site of the pCaβGFP expression vector (Alvares et al., 2003), creating a bi-cistronic construct.

#### *In ovo* electroporation

The occipital lateral mesoderm was electroporated at HH9-10^−^. A 2–5 mg/ml preparation of pCaβGFP was pressure-injected into the coelom, a 100 µm flame-sharpened tungsten wire was used as negative electrode and placed beneath the lateral mesoderm at the level of somite 1 or 1 and 2, a 500 µm platinum wire was used as positive electrode and placed above, two 20 ms/18 V rectangular pulses were then applied (Lours and Dietrich, 2005). Somites were electroporated at HH12 (occipital somites) or HH15 (wing-level somites), using a 2–5 mg/ml preparation of the pCaβGFP (control) or the Lbx1-Vp16 pCaβGFP construct. The flame-sharpened tungsten wire was used as negative electrode and placed inside the neural tube, the platinum wire was used as positive electrode and placed lateral to the somites, then one rectangular 20 ms/18 V pulse per somite was applied as described by Alvares et al. (2003).

#### *In ovo* microsurgery

Details of all microsurgical manipulation are provided in Supplementary Material 2.

#### Photomicroscopy

After completion of the staining reactions, embryos were cleared in 80% glycerol/PBS. Embryos and sections were photographed on a Zeiss Axioskop, using fluorescence or Nomarski optics.

## Results

### Dynamics of marker gene expression at the head–trunk interface

**Fig. 1:**
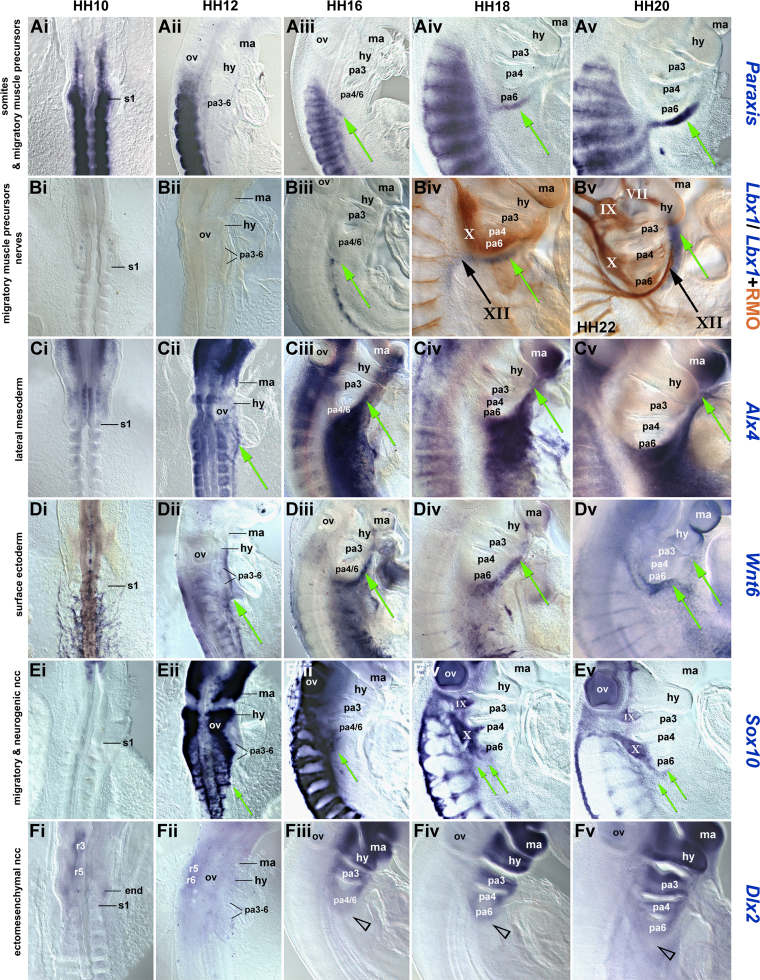
Coordinate rostral extension of marker gene expression domains at the chicken head–trunk interface. (Ai–Fi) dorsal, (Aii–Fii) dorsolateral and (Aiii–Fiii, Aiv–Fiv, Av–Fv) lateral views of the embryonic chicken head–trunk interface at the stages indicated at the top of the panel (Bv shows HH22), rostral to the top. The molecular markers are indicated on the right. Green arrows point at labelled cells using the circumpharyngeal route (black arrows for hypoglossal nerve). The expression domains of markers for the occipital lateral mesoderm (*Alx*4) and ectoderm (*Wnt*6) extend along the floor of the pharyngeal arches before this path is being used by the circumpharyngeal neural crest cells (*Sox*10 staining; *Dlx*2 labels the pharyngeal ectomesenchyme), the HMP (*Paraxis*, *Lbx*1 staining) and the hypoglossal nerve (RMO staining). Abbreviations: hy, hyoid arch; ma, mandibular arch; ncc, neural crest cells; ov, otic vesicle; pa3-6, pharyngeal arches 3-6; s1, 1st somite; VII, facial nerve; IX, glossopharyngeal nerve; X, vagal nerve; and XII, hypoglossal nerve.

**Fig. 2:**
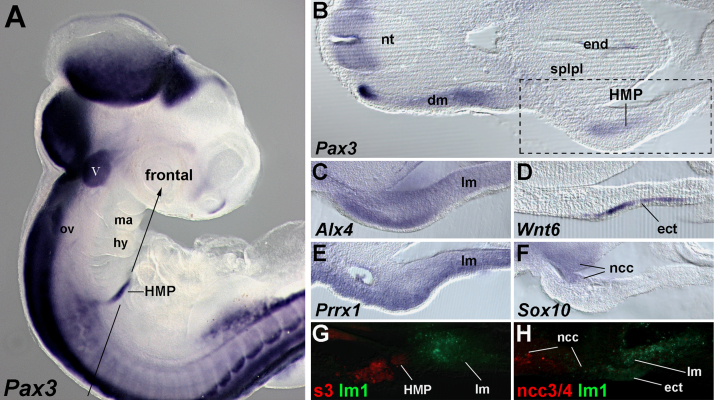
Position of occipital cells revealed by sections. (A) Lateral view of a HH19 chicken embryo stained for the expression of *Pax*3. (B–F) Sections of HH19 embryos, stained for the markers indicated, along the frontal sectional plane denoted in (A); dorsal to the left. (G, H) Corresponding sections of embryos that at HH9-10 had been DiO-injected into the lateral mesoderm and ectoderm next to somite 1 (lm1) and DiI-injected into somite 3 (G, s3) or the neural crest at the level of somites 3 and 4 (ncc3/4). Abbreviations: dm, somitic dermomyotome; ect, surface ectoderm; end, endoderm; hy, hyoid arch; lm, lateral mesoderm; ma, mandibular arch; ncc, neural crest cells; nt, neural tube; ov, otic vesicle; splpl, splanchopleure; and V, trigeminal ganglion.

To investigate which tissues at the head–trunk interface might move along the circumpharyngeal route, and in which order, we analysed the dynamics of marker gene expression, using *Paraxis* and *Pax*3 as markers for somites and migratory skeletal muscle precursors (Dietrich et al., 1998, 1997; Šošic et al., 1997), *Lbx*1 as marker exclusive for migratory muscle precursors (Dietrich et al., 1998), *Alx*4 and *Prrx*1 as lateral mesoderm markers (Takahashi et al., 1998; this study), *Wnt*6 as marker for the surface ectoderm (Schubert et al., 2002), *Sox*10 as a marker for migrating and neurogenic neural crest cells and *Dlx*2 as a ectomesenchymal neural crest cell marker (Blentic et al., 2008). Moreover, we traced the developing nervous system with the RMO-270 antibody that detects intermediate neurofilaments. We first traced expression from before the onset of any reported cell migration to the stage occipital cell populations are thought to be moving in the chicken embryo (Fig. 1). We then analysed the precise location of labelled cells on sections (Fig. 2). Finally, we performed a comparative expression analysis using models for sarcopterygian (mouse, chicken, and *Xenopus*) and actinopterygian (zebrafish) jawed vertebrates (Supplementary Material 3).

#### Somitic and HMP markers

*Paraxis* labelled the condensing somites and subsequently the somitic dermomyotomes at all stages (Fig. 1Ai–v; Šošic et al., 1997). Expression in HMP detaching from the occipital somites emerged at HH16 (Fig. 1Aiii, green arrow). At HH17-18, the *Paraxis* expressing cells coalesced into a single stream known as the hypoglossal cord (Fig. 1Aiv, green arrow; Ericsson et al., 2013). From HH18 onwards, HMP projected ventrolaterally and then rostrally along the floor of the pharynx towards the mandibular arch (Fig. 1Aiv and v, green arrows). *Pax*3 is known to label the primitive streak, neural tube, subpopulations of neural crest cells and the trigeminal ganglion (Goulding et al., 1993), somitic expression however showed the same dynamics as that of *Paraxis* (Fig. 2A, B, and not shown). *Lbx*1 expression in the somites commenced at HH15-16, exclusively labelling the hypaxial migratory muscle precursors (Fig. 1Biii and iv). Emigrating HMP expressing *Lbx*1 were seen from HH16 onwards; the cells continued to express *Lbx*1 in the same pattern as *Paraxis* or *Pax*3 (Fig. 1Biii–v, green arrows; Supplementary Fig. 3A). Double staining for *Lbx*1 (Fig. 1Biv and v, blue staining) and intermediate neurofilaments (Fig. 1Biv and v, brown staining) revealed that the outgrowth of the hypoglossal nerve, destined to innervate the hypoglossal muscles, lagged behind the emigrating muscle precursors, but the axons took the same route (Fig. 1Biv and v, arrow labelled XII).

#### Lateral mesoderm markers

*Alx*4 initially labelled the rostral non-somitic head mesoderm, the caudal hindbrain and weakly, the somites and mesencephalic neural crest cells (Bothe and Dietrich, 2006; Fig. 1Ci). At HH12, expression encompassed the trunk lateral mesoderm along the border to the intermediate and somitic mesoderm. The rostral expression boundary followed the contours of the caudal pharyngeal arches, thus adopting a curved shape (Fig. 1Cii, green arrow). From HH16 onwards, this expression intensified and extended rostrally along the floor of the pharyngeal arches, with the rostral limit significantly ahead of the emigrating hypoglossal muscle precursors (Fig. 1Ciii, iv, and I, green arrows; Supplementary Figs. 3B, arrows and 2C). At HH18-20, subpharyngeal *Alx*4 expression was well-established in the floor of the mandibular arch (which carried additional *Alx*4 expression in the distal neural crest cells (Qu et al., 1999)). *Prrx*1 expression in the occipital lateral mesoderm closely resembled that of *Alx*4 (shown for HH19 in Supplementary Figs. 3C and 2E).

#### Ectoderm markers

*Wnt*6 at HH10 labelled the surface ectoderm overlying the somites and the adjacent lateral mesoderm (Fig. 1Di). At HH12, the expression of *Wnt*6 extended ventrolaterally, avoiding the pharyngeal arches (Fig. 1Dii, green arrow). By HH16, *Wnt*6 expression had expanded rostrally along the pharynx (Fig. 1Diii, green arrow). At HH18-20, a similar pattern was found, with *Wnt*6 expression overlying the subpharyngeal expression of *Alx*4 (Fig. 1Div and v, green arrows; section shown in Fig. 2D).

#### Neural crest cell markers

*Sox*10 expression commenced in the cranial neural crest cells at HH9-10, and began to engulf the occipital region at HH12 (Fig. 1Ei and ii). At this stage, *Sox*10 strongly labelled a dorsolateral neural crest stream heading for the mandibular arch, a stream heading for the 2nd pharyngeal arch and a post-otic stream heading for the caudal pharyngeal arches. Moreover, cells that eventually will navigate around the arches had begun *Sox*10 expression and emigration (Fig. 1Eii, green arrow). By HH16 (Fig. 1Eiii), the neural crest cells inside the pharyngeal arches were *Sox*10-negative. Expression continued in the developing cranial and dorsal root ganglia, in cells using the ventrolateral path through the somites (except somites 1 and 2 (Ferguson and Graham, 2004)) and in cells caudal to the pharyngeal arches (Fig. 1Eiii, green arrow). This expression was maintained at HH18 and HH20; specifically, *Sox*10 expression was found in neural crest cells at and caudal to the circumpharyngeal ridge and in cells populating the enteric system (Fig. 1Eiv and v, green arrows and Fig. 2F). *Dlx*2 between HH10-12 transiently labelled rhombomeres 3 and 5–6 (Fig. 1Fi, ii; r3, 5, 6). At HH16-20, the gene was expressed in the ectomesenchymal neural crest cells that had filled the pharyngeal arches (Fig. 1Diii–v) while cells immediately caudal to the arches were *Dlx*2 negative (Fig. 1Fiii–v, open arrowhead).

Taken together, from HH12 onwards, occipital marker gene expression expanded along the same route along the pharynx, with the lateral mesoderm and ectoderm markers spreading first, followed by markers for HMP, circumpharyngeal neural crest cells and the hypoglossal nerve. Mouse, *Xenopus* and zebrafish showed corresponding expression patterns, pointing towards an evolutionarily conserved process (Supplementary Fig. 3).

### Mapping of cell movements at the head–trunk interface

Directionality and sequence of marker gene expansion suggest that occipitally derived cells undertake orchestrated cell movements. To corroborate this finding, we systematically labelled occipital tissues with fluorescent dyes, both in the chicken (amniote) and in the frog (anamniote). In the chicken, labellings were carried out at HH8+ to HH10−, before the onset of any reported cell movements and before the onset of any circumpharyngeal marker expansion. The position of the labelled cells was then recorded up to HH20-23, when lateral mesoderm, ectoderm, hypoglossal muscle precursor and nerve markers are well expressed along the floor of the pharyngeal arches. Labellings in the frog were performed at st 20 before the onset of *lbx*1 expression, and analysed 3 days later at st36-37. We used the actin:GFP line in which the developing somites are marked by GFP expression (Latinkic et al., 2002).

#### Labelling experiments in the chicken

##### Tracing of the occipital lateral mesoderm and ectoderm with DiI/DiO (*n*=15; Fig. 3)

**Fig. 3:**
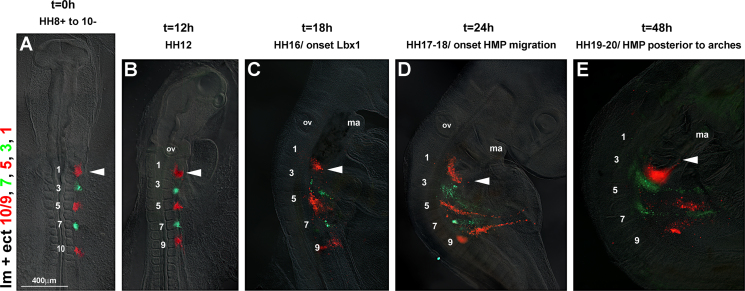
DiI/DiO labelling of the occipital lateral mesoderm and ectoderm reveals extensive cell movements. Dorsal (A, B) or lateral (C–E) views of the chicken head–neck interface at the stages indicated on top of the panel, rostral to the top. In each, the lateral mesoderm and overlying ectoderm next to somites 1, 3, 5, 7, and 9/10 (lm+ect 1, 3, 5, 7, and 9/10) had been labelled at stages HH8+ to HH10− with DiI (red) and DiO (green) as shown in (A). As development proceeded, the labelled cell groups became compressed rostrocaudally and stretched mediolaterally. Cell groups next to somite 1 (arrowhead) took the circumpharyngeal route, eventually extending rostrally along the floor of the arches and towards the mandibular arch; cells groups labelled next to somites 3–10 spread laterally–caudally. The cell movements matched the extension of *Alx*4 and *Wnt*6 expression shown in Fig. 1. Abbreviations: ma, mandibular arch and ov, otic vesicle.

The occipital and upper cervical lateral mesoderm and overlying ectoderm were injected with DiI and DiO next to somites 1, 3, 5, 7, and 9/10 in an alternating pattern (Fig. 3A). Twelve hours after labelling, the fluorescent cells groups had begun to expand in a lateral direction, with the cells originating from next to somite 1 forming a crescent (Fig. 3B, arrowhead). Eighteen hours after dye injection, the labelled cells groups had expanded further laterally (Fig. 3C). Cells labelled next to somites 3–9/10 formed stripes projecting laterally–caudally. In contrast, cells labelled next to somite 1 projected rostrally towards the floor of the pharyngeal arches (arrowhead). This pattern was more pronounced 24 hours after labelling (Fig. 3D). Forty-eight hours after labelling (Fig. 3E), the stripe originating from next to somite 1 and the more caudal stripes were clearly pointing into opposite directions. The stream of cells originating from next to somite 1 coincided with of the subpharyngeal expression of *Alx*4, *Prrx*1 and *Wnt*6 (compare Figs. 3, 1C and D; Supplementary Figs. 3B, C and 2C–E, G, H), suggesting that the expansion of gene expression domains was due to cells moving along the pharynx.

##### Tracing the rostral occipital lateral mesoderm using pCaβGFP electroporation (*n*=25; Fig. 4)

**Fig. 4:**
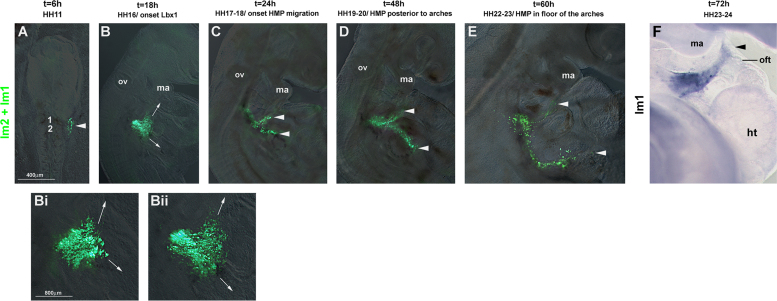
Electroporation of pCaβGFP confirms the extensive movement of the occipital lateral mesoderm. The lateral mesoderm next to somites 1-2 (A–E, lm1+lm2) or somite 1 (F, lm1) was electroporated with pCaβGFP at HH10. At the time points indicated on top of the panel, the electroporated cells were traced based on the fluorescence of GFP (A–E) or GFP mRNA expression (F, blue staining). Dorsal (A) or lateral (B–F) views, rostral to the top; (Bi, ii) are higher magnifications of labelled cells at HH16, Bii corresponds to the specimen shown in (B). Note that the lateral mesodermal cells derived from somite levels 1 and 2 organised themselves into two streams, one extending along the floor of the pharyngeal arches towards the mandibular arch and the outflow tract of the heart, the other one extending laterally. Abbreviations: ht, heart; ma, mandibular arch; oft, outflow tract of the heart; and ov, otic vesicle.

Fluorescent dyes dilute out quickly due to cell division. To obtain more robust labellings, we electroporated the occipital lateral mesoderm (somatopleura) with the GFP expressing vector pCaβGFP (Alvares et al., 2003), focussing on the region next to somites 1 and 2. The construct was expressed 6 hours after electroporation, labelling a compact cell group (Fig. 4A, arrowhead). Eighteen hours after electroporation, this cell group had expanded laterally (Fig. 4B). At higher magnification, cells could be seen to organise themselves into two streams, one heading rostrally and one heading in a lateral–caudal direction (Fig. 4Bi and ii). Twenty-four hours after electroporation, the cell group had split into two streams, the rostral stream projecting rostrally towards the floor of the pharyngeal arches. Forty-eight hours and 60 hours after electroporation, the separation of the two labelled cell groups was more pronounced, with the rostral stream of cells travelling rostrally along the floor of the pharyngeal arches. Notably, the rostral stream of electroporated cells followed the same trajectory as the DiI labelled cells from next to somite 1, coincided with the position of *Alx*4 and *Prrx*1 expressing cells (compare Figs. 3 and 4, 1C; Supplementary Figs. 3B, C and 2C, E, G, H), and reached as far rostrally as the floor of the mandibular arch and the outflow tract of the heart.

##### Tracing the hypopharyngeal/hypoglossal muscle precursors (HMP) (*n*=13; Fig. 5)

**Fig. 5:**
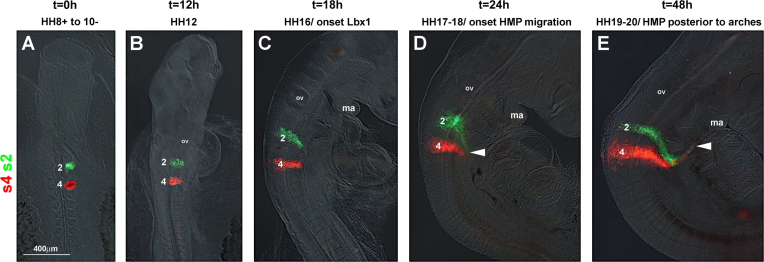
DiI/DiO labelling of hypobranchial/hypoglossal muscle precursors (HMP). Dorsal (A, B) or lateral (C–E) views of chicken embryos, rostral to the top. The occipital somites number 2 and 4 (s2, s4), both know to contribute to the hypobranchial/hypoglossal musculature, were labelled with DiI (red) and DiO (green) at HH8+ to HH10, and the position of these cells was recorded at the time points indicated on top of the panel. The labelled cells all collected in a single point at HH17-18 and travelled first ventrolaterally, then rostrally in a single stream. Abbreviations: ma, mandibular arch and ov, otic vesicle.

Chicken HMP are known to stem from occipital somites 2–5/6 (Couly et al., 1993; Huang et al., 1999; Noden, 1983; Wachtler and Jacob, 1986). To monitor when these somites release cells into the hypoglossal cord, somite 2 was injected with the DiO and somite 4 with DiI at stages HH8+ to HH10− (Fig. 5A). Twelve to eighteen hours after dye injection, labelled cells were still within the confinement of the somite (Fig. 5B and C). Twenty-four hours after labelling, fluorescent cells were seen lateral to the somites with cells collecting in a single point (Fig. 5D, arrowhead). Forty-eight hours after labelling, the fluorescing cells had formed a condensed stream of intermingled red and green cells that surrounded the caudal pharyngeal arches and headed rostrally towards the floor of the mouth (Fig. 5E, arrowhead). The position of the labelled cells corresponded to the position of the *Paraxis*, *Pax*3 and *Lbx*1 expressing cells of the hypoglossal cord (compare Fig. 5 and Figs. 1 and 2).

##### Tracing the HMP in comparison to the occipital lateral mesoderm (*n*=24; Fig. 6)

**Fig. 6:**
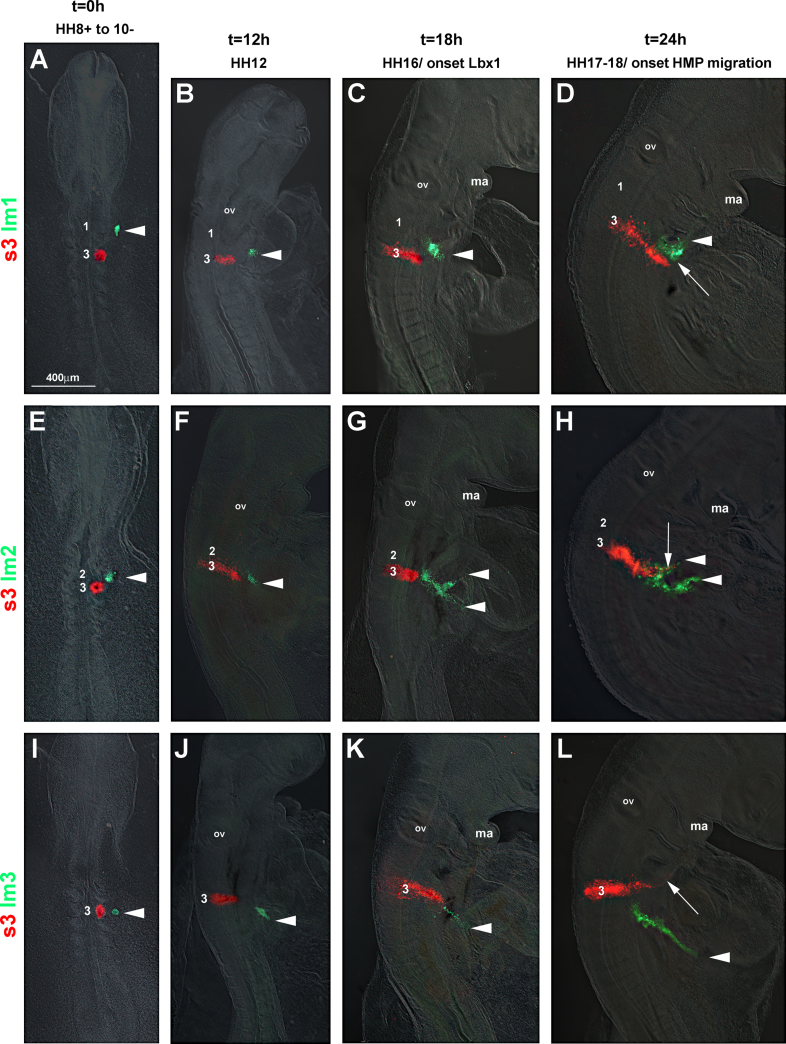
DiI/DiO mapping of HMP versus lateral mesoderm cell movements. Dorsal or lateral views of chicken embryos as in Fig. 5, with somite 3 (s3) labelled with DiI and the lateral mesoderm next to somite 1 (lm1; A–D), next to somite 2 (lm2; E–H) or next to somite 3 (lm3; I–L) labelled with DiO. The lateral mesoderm cell groups begin their morphogenetic movements before the deepithelialisation of TMP, with cells originally situated next to somite 1 (C, D; arrowhead) leading the TMP (C, D; arrow). The lateral mesodermal cell group originally found next to somite 2 split into two streams (G, H; arrowheads); the TMP are embedded in the rostral of these two streams (H, arrow). Lateral mesoderm originating from a position next to somite 3 are already displaced ventrolaterally when the TMP begin to head towards the floor of the pharyngeal arches (K, L). Abbreviations: ma, mandibular arch and ov, otic vesicle.

To map the relative movement of the HMP and the occipital lateral mesoderm, we labelled somite 3, one of the somites contributing to the hypoglossal cord, with DiI and the lateral mesoderm next to somite 1 (Fig. 6A–D), next to somite 2 (Fig. 6E–H) or next to somite 3 (Fig. 6I–L) with DiO. Twelve hours after labelling, while the position of the somitic cells was unchanged, the lateral mesoderm cells were displaced caudally (compare position of red and green label in Fig. 6A, E, and I versus B, F, and J), with cell groups appearing rostrocaudally compressed and ventrolaterally expanded, confirming that coordinated cell movements were already under way. Eighteen hours after labelling, lateral mesoderm that originated next to somite 1 had formed a stream of cells that surrounded the pharyngeal arches (Fig. 6C, arrowhead). The lateral mesoderm labelled next to somite 2 had split into two streams (Fig. 6G, arrowheads). The lateral mesoderm that once resided next to somite 3 had formed a stream of cells heading in a lateral–caudal direction (Fig. 6K, arrowhead). In contrast, the somitic cells had just begun to leave the somite. Twenty-four hours after dye injection, HMP began to move ventrally–rostrally, in line with *Paraxis*/*Pax*3/*Lbx*1 staining (Fig. 6D, H, and L; arrows). The cells followed the stream of lateral mesoderm from next to somite 1 (Fig. 6D, arrowhead; section shown in Fig. 2G) and were embedded in the rostral of the two streams originating from next to somite 2 (Fig. 6H, upper arrowhead). Thus, a dynamic pattern of cell movement is established at the head–trunk interface, with a rostral stream of lateral mesoderm cells engulfing and leading the HMP.

##### Tracing the neural crest cells in comparison to the occipital lateral mesoderm (*n*=29; Fig. 7)

**Fig. 7:**
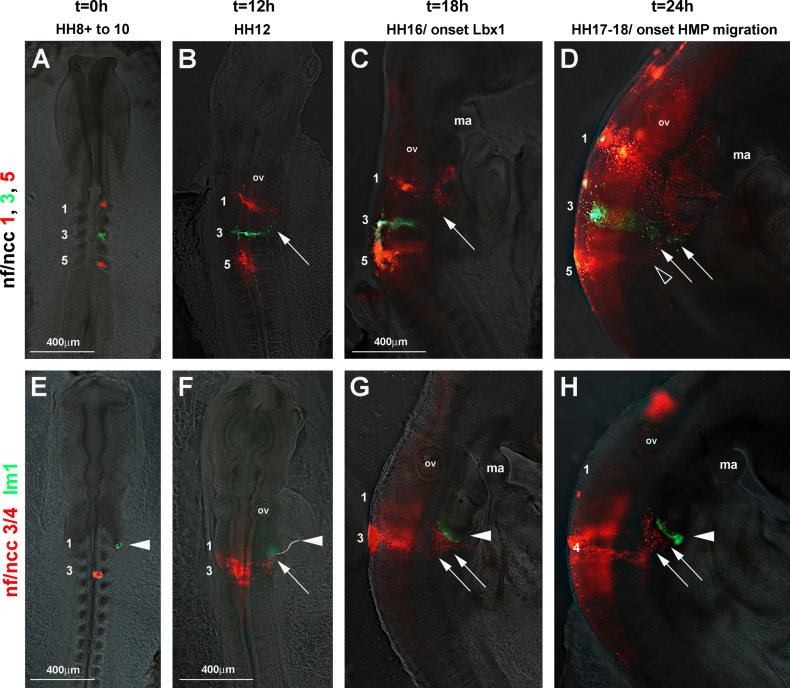
DiI/DiO mapping of occipital neural crest cell versus lateral mesoderm cell movements. Dorsal or lateral views of chicken embryos as in Fig. 5. (A–D) Tracing of neural crest cells (ncc). (A) Labelling of the neural folds overlying somites 1, 3, and 5 with DiI and DiO at HH8. (B) At HH12, nnc from the levels of somites 1 and 3 are actively migrating laterally; cells from the level of somite 5 have started their migration. (C, D) At HH16-18, ncc from somite level 1 fill the caudal pharyngeal arches, ncc from the level of somite 3 navigate around the most caudal arch (arrows), ncc from somite level 5 remain caudal to the ncc population from somite level 3 (open arrowhead). (E–H) Tracing of ncc originating from somite levels 3–4 with DiI and lateral mesoderm from somite 1 levels with DiO. (E) Labelling of cells at HH8-10. (F) At HH12, the lateral mesodermal cells have moved laterally–ventrally (arrowhead), the ncc are following behind (arrow). (G, H) At HH16-18, the lateral mesodermal cells move rostrally along the floor of the pharynx (arrowheads), closely followed by the ncc (arrowheads). Abbreviations: ma, mandibular arch and ov, otic vesicle.

To establish the relative movement of occipital neural crest cells and lateral mesoderm, we first retraced the occipital crest, focusing on cells originating from somite levels 1, 3, and 5 (Fig. 7A–D). In line with the lateral expansion of *Sox*10 expression, all labelled neural crest cell groups were emigrating at HH12 (Fig. 7B), with cells derived from somite 1 and 3 levels having progressed further than cells from the level of somite 5. Subsequently, cells originating from the level of the 1st somite populated the caudal pharyngeal arches; cells originating from the level of somite 3 populated the territory caudally adjacent to the arches (Fig. 7B–D, arrows) and cells from the level of the 5th somite settled caudal to the cells derived from somite level 3 (Fig. 7D, open arrowhead). Thus, it is neural crest cells from the level of somites 3 and 4 that migrate into the area also used by the rostrally projecting lateral mesoderm and the HMP. Next, we labelled neural crest cells at the level of somites 3 and 4 with DiI and the lateral mesoderm at the level of somite 1 with DiO as shown in Fig. 7E. After 12 hours, the lateral mesoderm had translocated into a more ventral position (Fig. 7F, arrowhead) with the neural crest cells following behind (Fig. 7F, arrow). After 18 hours (the time that HMP begin *Lbx*1 expression), and after 24 h (the time HMP begin to emigrate) lateral mesoderm (Fig. 7G and H, arrowhead) and neural crest cells (Fig. 7G and H, arrows; section shown in Fig. 2H) were surrounding the caudal-most pharyngeal arch. Thus, of the occipital cells participating in the ventrally–rostrally directed movement, the lateral mesoderm was ahead of the neural crest cells which in turn were slightly ahead of the cells from the paraxial mesoderm.

#### Labelling experiments in the frog (*n*=13, Fig. 8)

**Fig. 8:**
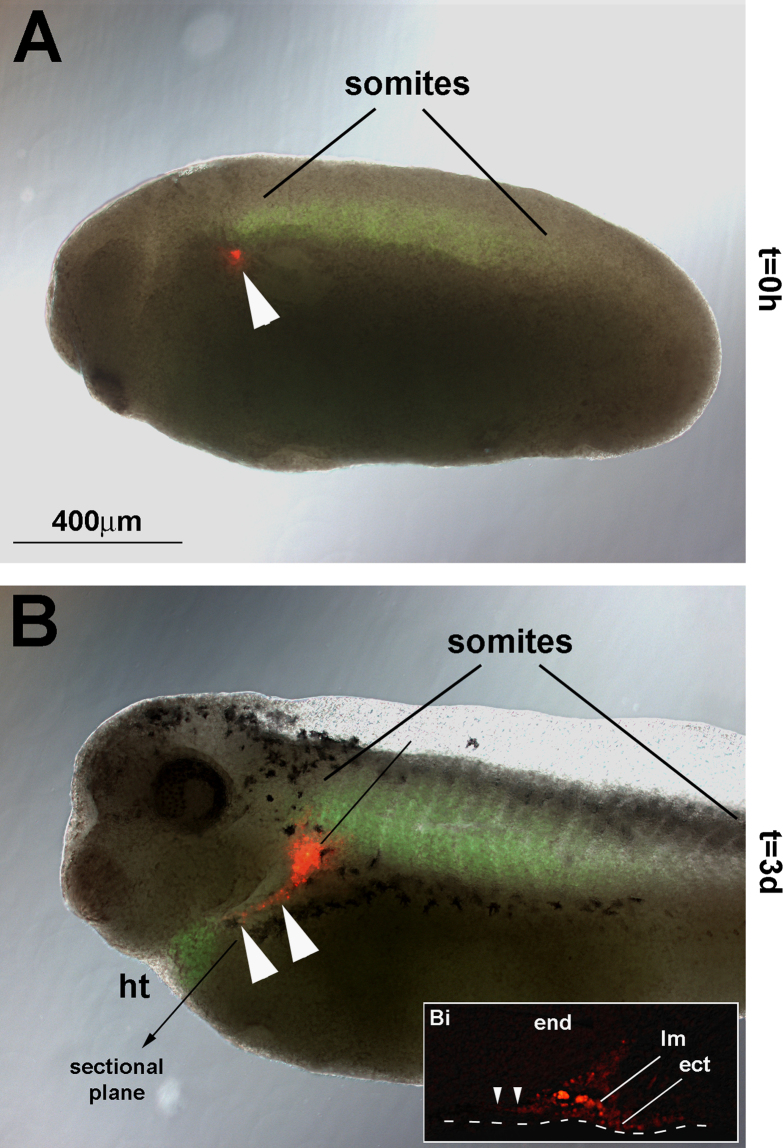
DiI labelling of the *Xenopus* occipital lateral mesoderm. (A, B) Lateral views of *Xenopus laevis* transgenic cardiac actin:GFP embryos, rostral to the left, dorsal to the top. (Bi) Section of the embryo shown in (B) along the plane indicated in (B), dorsal to the right, medial to the top. (A) DiI-labelling (arrowhead) of the occipital lateral mesoderm next to the 1st somite (as identified by its green fluorescence) at st 20. (B, Bi) Three days later, the labelled lateral mesoderm has expanded in a ventral–rostral direction, surrounding the pharyngeal arches and heading towards the floor of the arches (arrowheads) as previously seen for the chicken. Abbreviations: ect, surface ectoderm; end, endoderm; ht, heart; and lm, lateral mesoderm.

To investigate whether the cell movements found in the chicken are also present in anamniotes, we labelled the occipital lateral mesoderm next to somite 1 in actin:GFP frogs with DiI (Fig. 8A). Three days later, the labelled cells had formed a crescent surrounding the pharyngeal arches in the same manner as seen for the chicken (Fig. 8B and Bi, arrowheads). Taken together, our expression data and labelling experiments suggest that coordinated cell movements at the head–trunk interface are a common feature of osteichthyans, possibly of all vertebrates.

### Significance of the cell movements at the head–trunk interface

**Fig. 9:**
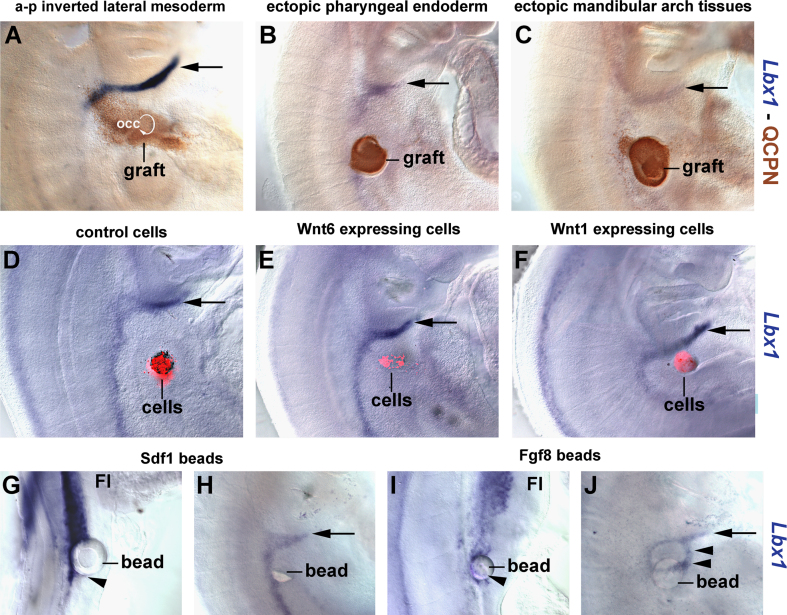
Challenging HMP with putative attractants. Lateral views of chick embryos 36 hours after surgery at HH18-19, rostral to the top, lateral to the right. *Lbx*1 expression is displayed in blue, quail-derived grafts in brown (A–C) and grafted cell pellets are marked with Celltracker Orange in red (D–F). (A) Rostrocaudal inversion of the occipital lateral mesoderm and ectoderm or (B) insertion of pharyngeal endoderm next to the caudal-most occipital somite or (C) insertion of mandibular arch mesenchyme plus overlying oral epithelium next to caudal-most occipital somites all do not divert TMP from their usual path (arrows). (D) Implanting RatB1 control cells next to the caudal-most occipital somite or elevating canonical Wnt signalling at this position by implanting cells secreting Wnt6 (E) or Wnt1 (F) also does not redirect HMP. (G, H) Implantation of Sdf1 loaded beads. Migratory limb muscle precursors at the caudal boundary of the limbs accumulated around the bead (G, arrowhead). (H) Sdf1-bead implantation next to the caudal-most occipital somites; HMP followed their normal path (arrow). (I, J) Implantation of Fgf8 loaded beads. When implanted at the interface of limb and flank level somites, the bead triggered the formation and emigration of migratory muscle precursors (I, arrowhead). (J) When implanted next to the caudal-most occipital somites, HMP were temporarily attracted by the bead (arrowheads) and then continued with their normal path (arrow). Thus, overriding forces prevent HMP deviation from their circumpharyngeal path. Abbreviations: FL, fore limb and occ, occipital lateral mesoderm.

**Fig. 10:**
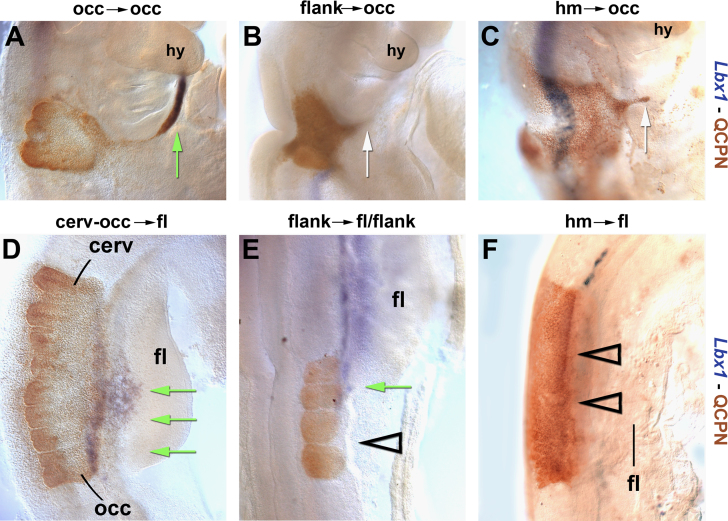
Behaviour of non-migratory muscle precursors grafted to the occipital region. Lateral views of chick embryos at HH18-19, 36 hours after surgery; *Lbx*1 expression shown in blue, quail cells in brown; rostral is to the top, lateral to the right. Migratory muscle precursors expressing *Lbx*1 and entering the lateral mesoderm are marked with green arrows; white arrows indicate cells entering the lateral mesoderm without expressing *Lbx*1, open arrowheads mark cells that neither express *Lbx*1 nor trespass into the lateral mesoderm. (A) Control grafting of quail occipital somites in place of chick occipital somites. The quail cells express *Lbx*1 and leave the somites, contributing to the hypoglossal cord and eventually forming hypobranchial/hypoglossal muscle (green arrow). (B, C) Grafting of quail flank level somites (B) or quail head mesoderm (C) in place of chick occipital somites. The quail cells do not have characteristics of migratory muscle precursors as they lack expression of *Lbx*1. However, they enter the lateral mesoderm along the route normally taken by TMP (white arrow). (D) Control grafting of quail occipital and upper cervical somites in place of chick somites at forelimb levels. The quail cells express *Lbx*1 and migrate into the limb (green arrow), mimicking limb muscle precursors. (E) Grafting of quail flank somites in place of chicken somites at the forelimb–flank boundary. Cells under the influence of the forelimb become reprogrammed, expressing *Lbx*1 and contributing to the limb musculature (green arrow). Cells exposed to flank cues do not express *Lbx*1 or emigrate (open arrowhead). (F) Grafting of quail head mesoderm in place of chicken forelimb somites as shown in (Mootoosamy and Dietrich, 2002). The cells neither express *Lbx*1 nor migrate into the limb, indicating that they are not converted into migratory muscle precursors (open arrowhead). These findings suggest that in the occipital region, non-migratory cells may undertake movements similar to HMP. Abbreviations: cerv, cervical somites; fl, forelimb; hm, head mesoderm; hy, hyoid arch; and occ, occipital somites.

**Fig. 11:**
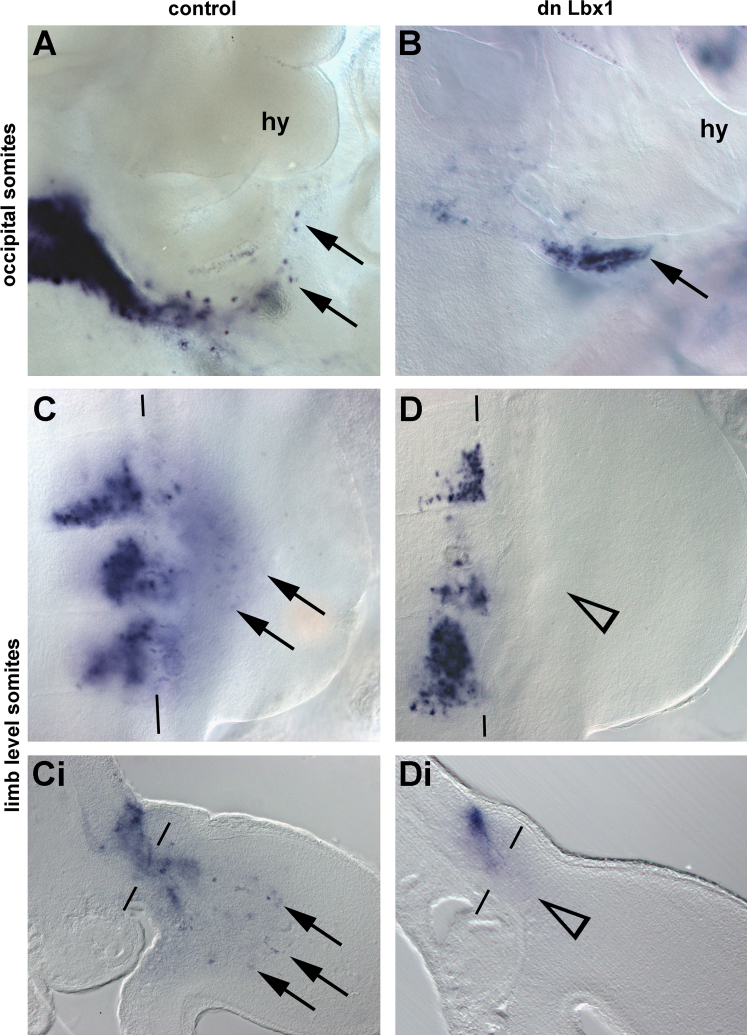
Behaviour of muscle precursors electroporated with a dominant negative *Lbx*1 construct. (A–D) Lateral views, rostral to the top, and (Ci, Di) cross sections, dorsal to the top, of somites electroporated with the pCaβGFP control vector (A, C, Ci) or the vector encoding a dominant negative (dn) *Lbx*1*-VP*16 construct (B, D, Di). Cells harbouring the constructs are shown in blue; the lateral edges of somites are indicated by black lines in (C, Ci, D, Di). Cells from the lateral edge of control-electroporated somites emigrated normally, both at occipital (A, arrows) or at forelimb levels (C, Ci; arrowheads). When the *dnLbx*1 construct was used, muscle precursors at forelimb levels failed to emigrate (D, Di; open arrowheads). In contrast, occipital muscle precursors, albeit delayed, projected along the normal path of HMP (B, arrow). Abbreviations: hy, hyoid arch.

**Fig. 12:**
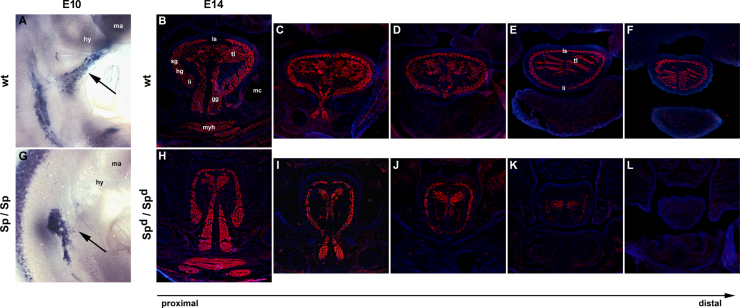
Hypoglossal/hypobranchial muscles develop in the *Pax*3 mouse mutant *Splotch delayed*. (A, G) Wildtype and *Splotch* (*Sp*/*Sp*) mutant embryos at E10 of development, stained for the expression of *Lbx*1, lateral view, rostral to the top. The hypoglossal cord containing the emigrating HMP is well developed in the wildtype (A, arrow); in the mutant, cells projecting towards the floor of the pharyngeal arches are also visible (G, arrow). (B–F) Serial frontal sections from the proximal root (B) to the distal tip (F) of the tongue of a E14 wildtype mouse head, stained for Myosin Heavy Chains. Intrinsic and extrinsic hypoglossal muscles are well developed. (H–L) Corresponding frontal sections of a E14 *Splotch delayed* (*Sp*^*d*^/*Sp*^*d*^) head; although reduced, hypoglossal muscles developed. Abbreviations: fl, fore limb; gg, genioglossus muscle; hg, hyoglossus muscle; hy, hyoid arch; li, longitudinalis inferior muscle; ls, longitudinalis superior muscle; ma, mandibular arch; mc, Meckel׳s cartilage; myh, mylohyoid muscle; sg, styloglossus muscle; tl, transverses linguae muscle; and wt, wildtype.

The rostrally directed, hypopharyngeal cell movements spearheaded by the lateral mesoderm may create forces to drag later emerging cell populations along. To test this, we focused on HMP. We first established in the chicken that the occipital somites behave like somites further caudally in the trunk (Supplementary Material 4). We then grafted putative attractants to deviate HMP (Fig. 9), we replaced HMP with non-migratory muscle precursors (Fig. 10) and we molecularly rendered HMP non-migratory (Fig. 11). Finally, we investigated hypoglossal muscle formation in the mouse mutant *Pax*3^*Sp*^ (Fig. 12). Graftings were carried out at HH9-10 (controls at fore limb levels: HH15) and analysed at HH18-19 (details of these procedures: Supplementary Material 4). HMP cells were traced by the means of *Lbx*1 expression (Figs. 9–11, blue staining).

#### Challenging the HMP with putative attractants

##### Rostrocaudal inversion of the occipital lateral mesoderm

The first target of emigrating HMP is the neighbouring occipital lateral mesoderm. To test whether this tissue guides the HMP rostrally, we grafted the left occipital lateral mesoderm from quail to the right side of a chicken host, such that the rostrocaudal orientation of the graft was inverted but the mediolateral orientation was unchanged (*n*=5). Thirty-six hours later at HH18-19, the graft (detected with the QCPN antibody; Fig. 9A, brown staining) was compressed in rostrocaudal and expanded in mediolateral direction, in line with our earlier labelling experiments. The HMP (Fig. 9A, arrow) were not deviated and projected along the normal circumpharyngeal route.

##### Grafting of pharyngeal endoderm

The floor of the pharynx is the second target of HMP migration. We explanted quail pharyngeal endoderm before HMP pass along the pharynx at HH15-16 and inserted this tissue next to the caudal-most occipital somites of a chicken host (*n*=12; Fig. 9B, brown staining). We expected that the HMP would turn towards the implant if this provided attracting cues. However, the HMP followed their normal path (Fig. 9B, arrow).

##### Grafting of mandibular arch tissues

The floor of the mandibular arch is the final target of HMP migration. We explanted mandibular arch mesenchyme and oral ectoderm from HH23 quail embryos (the stage before the HMP reach the floor of the mouth) and, as before, implanted the tissues next to the caudal-most occipital somites of a chicken host. However, the HMP again ignored the implant (*n*=7; Fig. 9C, arrow).

##### Grafting of Wnt expressing cells

The ectoderm overlying the occipital region and the path of the HMP expresses the signalling molecule Wnt6 (Schubert et al., 2002; Figs. 1 and 2). Thus, we tested whether ectopic sources of Wnt signalling molecules may divert HMP from their normal path. We implanted CellTracker Orange stained RatB1 control cells (*n*=9; Fig. 9D) or RatB1 cells expressing mouse Wnt6 (*n*=8; Fig. 9E) or RatB1 cells expressing the in our hands more potent Wnt1 ligand (*n*=5; Fig. 9F (Cheng et al., 2004)) next to the caudal-most occipital somites. Moreover, we tested NIH3T3 or DF1 cells transfected with a pCAb or RCAS-based expression construct for mouse Wnt6 (*n*=10, not shown). None of these manipulations deviated the HMP from their course (Fig. 9D–F, arrows).

##### Grafting of Sdf1 beads

The chemokine Stromal cell derived factor 1/C-X-C motif ligand 12 (Sdf1/CXCL12) has been suggested to synergise with Scatter Factor/Hepatocyte growth factor (SF/HGF) to guide migratory limb muscle precursors into the limb (Vasyutina et al., 2005). In the chicken, SF/HGF is not expressed along the path of HMP (Mackenzie et al., 1998), but Sdf1 has been suggested as potential regulator of HMP emigration (Vasyutina et al., 2005). We therefore implanted beads loaded with Sdf1 next to the somites at the forelimb–flank boundary as control (*n*=7) or next to the caudal-most occipital somites (*n*=3), and assayed for the accumulation of *Lbx*1 expressing muscle precursors around the bead. This accumulation was seen in 4/7 control embryos (Fig. 9G, arrowhead), but not in any of the embryos where the beads were grafted occipitally (Fig. 9H, normal path of HMP marked by arrow).

##### Grafting of Fgf8 beads

Fibroblast growth factors Fgf4 and Fgf8 produced by the apical ectodermal ridge of the developing limb attract muscle precursors into the limb (Alvares et al., 2003). Occipital somites are patterned like trunk somites (Supplementary Material 1) and when grafted to the forelimb region, migrate into the limb like the original limb muscle precursors (see below). Therefore, we examined whether Fgf8 loaded beads may be able to deviate HMP from their normal path. In 5/6 control experiments, Fgf8 beads placed at the forelimb–flank boundary recruited the flank lateral mesoderm for limb development, overwrote the original programs in the flank somites and attracted *Lbx*1 expressing muscle precursors (Fig. 9I, arrowhead). When implanted at occipital levels, Fgf8 in 1/2 cases deviated the HMP (Fig. 9J, arrowheads), but eventually the cells progressed along the normal circumpharyngeal path (Fig. 9J, arrow).

#### Behaviour of migration-incompetent muscle precursors at the head–trunk interface

##### Grafting of non-migratory muscle precursors to the occipital region

The previous experiments suggested that the moving occipital tissues might provide forces that keep HMP on their circumpharyngeal path. We next asked whether these forces would be sufficient to carry non-migratory muscle precursors along. To test this, in a first series of experiments we replaced the chicken occipital somites with quail occipital somites (control 1), limb level somites (control 2; not shown), flank somites or with non-somitic head mesoderm. As further controls, we replaced limb level somites with these grafts. Details of the graftings are shown in Supplementary Material 2. Of the transplanted tissues, occipital and limb level somites normally produce migratory muscle precursors; flank somites do not but can do so in a limb environment (Alvares et al., 2003). The head mesoderm in contrast is unable to read trunk-specific cues and to provide migratory limb muscle precursors (Mootoosamy and Dietrich, 2002). Embryos were all cultured to HH18-19, analysed for the expression of *Lbx*1 (Fig. 10, blue staining) and, tracing the grafted quail cells with the QCPN antibody, for cell migration into the periphery (Fig. 10, brown staining).

When occipital (*n*=4; Fig. 10A) or limb level somites (*n*=2; not shown) were grafted to occipital levels, they released *Lbx*1 expressing muscle precursors into the hypoglossal cord (green arrow). Likewise, occipital (*n*=8; Fig. 10D) or limb level somites (*n*=6; not shown) grafted to limb levels released a broad front of *Lbx*1-positive cells into the limbs (green arrows). When flank somites were grafted to limb levels, these somites were reprogrammed to release *Lbx*1 expressing migratory muscle precursors (*n*=6; Fig. 10E, green arrow (Alvares et al., 2003)). When grafted to occipital levels, flank somites were not reprogrammed to produce *Lbx*1 expressing cells (*n*=2; Fig. 10B). However, the cells formed a triangularly shaped protrusion that extended along the route normally occupied by the hypoglossal cord (white arrow, in line with similar observations by (Mackenzie et al., 1998)). When the non-somitic head mesoderm was grafted to limb levels, this tissue did not express any somitic markers including *Lbx*1, and did not send migratory cells into the limb buds (*n*=12; Fig. 10F, open arrowheads (Mootoosamy and Dietrich, 2002)). When grafted to occipital levels, the head mesoderm also failed to express *Lbx*1 (*n*=5; Fig. 10C). However, again a triangularly shaped protrusion along the path normally taken by HMP formed (white arrow). This suggests that in the occipital environment, cells lacking the properties of migratory muscle precursors nevertheless are able to participate in movements typical for HMP.

##### Rendering the chicken HMP non-migratory

In the next series, we directly rendered HMP non-migratory, introducing a *Lbx*1*-Vp*16 pCaβGFP construct. This construct delivers a dominant negative form of *Lbx*1 since the EH transrepression domain of *Lbx*1 was replaced by the VP16 transactivating domain, thus activating all genes normally repressed by *Lbx*1. The construct was electroporated into occipital somites at HH12 or, as control, into forelimb level somites at HH15. As further controls, the empty pCaβGFP vector was electroporated. Embryos were analysed at HH18-20, tracing the position of the targeted cells by means of GFP fluorescence (not shown) and by whole mount in situ hybridisation with a GFP probe (Fig. 11, blue staining). When the pCaβGFP control construct was electroporated, cells from the occipital somites contributed to the hypoglossal cord in 9/16 cases (Fig. 11A, arrows), and cells from the limb-level somites migrated into the limb in 4/4 cases (Fig. 11C and Ci, arrows). When the Lbx1-Vp16 construct was electroporated into the limb-level somites, the electroporated cells failed to migrate into the limb in all cases (*n*=20; Fig. 11D and Di, open arrowhead). When the Lbx1-Vp16 construct was electroporated into occipital somites, in 4/18 cases cells were found well down the normal path of HMP (Fig. 11B, arrow). This suggests that at occipital but not at limb levels, non-migratory muscle precursors have a chance to be dragged along when captured by the stream of moving cells.

##### Behaviour of migration-incompetent HMP in the mouse mutant Splotch delayed

In electroporation experiments, typically not all cells take up the construct. Therefore, it cannot be excluded that the cells expressing *dnLbx*1 may have been dragged along by the wildtype HMP. We therefore turned to the *Pax*3 mouse mutant *Splotch delayed. Pax*3 is an upstream regulator of *cMet* and *Lbx*1, and in the mutant all migratory muscle precursors are deficient and a defined hypoglossal cord is absent at days E9.5–11.5 of embryonic development (Dietrich et al., 1999; Tremblay et al., 1998). We investigated the mutant, using a *Lbx*1 probe for whole mount in situ hybridisation at E10 and a cocktail of antibodies detecting sarcomeric Myosins on frontal sections at E14.

In wildtype controls at E10, *Lbx*1 expressing HMP had collected into the hypoglossal cord and reached the floor of the pharyngeal arches (Fig. 12A, arrow). At E14, anatomically well-defined hypobranchial and hypoglossal muscles were present (Fig. 12B–F, red staining). In the E10 *Sp*^*d*^/*Sp*^*d*^ mutant, the hypoglossal cord was absent, but a triangular protrusion of cells along the normal HMP path was visible (Fig. 12G, arrow). At E14, the mutant had a smaller tongue with less overall muscle mass. However, hypoglossal muscles were clearly recognisable (Fig. 12H–L, red staining). This suggests that, indeed, non-migratory muscle precursors have been dragged along by the surrounding cell types.

## Discussion

Deep in vertebrate evolution, the pharynx, originally only used for filter feeding, was adapted for respiration and active predation, and tissues originating from the trunk were recruited into the head to, amongst other functions, assemble an elaborate hypopharyngeal support system (reviewed in (Ericsson et al., 2013; Goodrich, 1958)). In osteichthyans, the hypopharyngeal/hypoglossal musculature develops from occipital muscle precursors that migrate along a circumpharyngeal path to reach their target site (reviewed by Gilbert (2000), Noden and Francis-West (2006), Dietrich et al. (1999, 1998), Kusakabe et al. (2011), Martin and Harland (2006), Neyt et al. (2000), Ochi and Westerfield (2009), Sabo et al. (2010), and Thisse et al. (2004); this study). Yet, the presence of hypopharyngeal muscles in cartilaginous and jawless vertebrates (Cole et al., 2011; Kuratani et al., 1999; Kusakabe et al., 2011; Neal, 1897; Neyt et al., 2000); reviewed in (Edgeworth, 1935; Ericsson et al., 2013; Goodrich, 1958) as well as in mouse mutants with defective migratory muscle precursors (Bladt et al., 1995; Dietrich et al., 1999; Prunotto et al., 2004; this study) suggests that there was and still is a traceable, ancestral mechanism of hypopharyngeal cell transport and organ assembly. The aim of this study was to discover what this mechanism might be. Our work suggests that morphogenetic cell movements spearheaded by the occipital lateral mesoderm and overlying ectoderm create forces to coordinate hypopharyngeal cell transport.

### Vertebrate occipital tissues undertake evolutionarily conserved cell movements that reach the floor of the mandibular arch and outflow tract of the heart

When investigating the expression of markers for paraxial and lateral mesoderm, neural crest cells, nerves and surface ectoderm at the head–trunk interface of various osteichthyans (chicken, mouse; frog representing sarcopterygians; zebrafish representing actinopterygians), we noticed that all markers showed a stereotype pattern of ventral and rostral expansion along the circumpharyngeal route. Moreover, markers for the lateral mesoderm and ectoderm were always in the lead. Labelling experiments showed that the dynamics of marker gene expression is due to cells moving around the pharyngeal arches and rostrally along the pharynx, with the lateral mesoderm reaching as far rostrally as the floor of the mandibular arch and outflow tract of the heart. Thus, occipitally derived cells and tissues undertake so far unknown, stereotype and conserved long-range movements. Currently, labelling data for the movement of occipital tissues in chondrichthyans and agnathans cannot readily be generated. However, in the lamprey markers labelling the hypobranchial/hypoglossal muscles and hypoglossal nerve show the same pattern as observed here (Kusakabe et al., 2011). This suggests that the trajectory of occipital cells and tissues was established before the gnathostome-agnathan split, and hence, the occipital cell movements may be a plesiomorph (=ancestral) vertebrate feature (Supplementary Fig. 5). Uro-, cephalo- and hemi-chordates lack the complicated muscularised pharyngeal apparatus of vertebrates, and it will be interesting to see if the movements of the lateral mesoderm and ectoderm around the pharyngeal apparatus prefigure the appearance of the muscularised vertebrate pharynx and the shift from filter feeding to respiration and active predation.

### The anterior stream of occipital lateral mesoderm cells leads and engulfs the circumpharyngeal crest, HMP and hypoglossal nerve

When labelling cells derived from distinct occipital levels in the chicken, we found that these cells partake in the newly discovered cell movements in a specific order and pattern. Cells originating from next to somite 1 navigate along the circumpharyngeal ridge and, when reaching the floor of the arches, proceed in a rostral direction. Cells originating from somite levels 3 to 5 project laterally–caudally. Cells originating from the level of the 2nd somite either follow the cells that originated from somite 1 levels rostrally, or alternatively, participate in the laterally–caudally directed movements, thus contributing to both streams. Paraxial mesodermal cells begin their movements when the lateral mesoderm is well on its way. Significantly, the hypobranchial/hypoglossal muscle precursors (HMP) originating from somites 2 to 5 collect in one point and then become embedded within the rostrally projecting lateral mesoderm derived from the level of the 2nd somite, with the lateral mesoderm from somite level 1 in the lead. Similarly, neural crest cells originating from somites 3 and 4 levels meet at the tail end of the rostrally projecting lateral mesoderm before navigating along the circumpharyngeal ridge, pursued by the outgrowing axons of the hypoglossal nerve. Thus, at all times, the lateral mesoderm and ectoderm stay in the lead and engulf the later emerging cell populations.

### The rostrally directed occipital cell movements underpin HMP movement

To explore the significance of the newly discovered cell movements, we challenged occipital muscle precursors in various ways. Yet all attempts to deviate HMP from their normal route failed – the cells always populated the circumpharyngeal path. Moreover, non-migratory cells derived from flank somites or from the non-somitic paraxial head mesoderm also populated this path albeit delayed. HMP rendered non-migratory via electroporation of a *dnLbx*1 construct in the chicken or due to a mutation of the upstream regulator *Pax*3 in the mouse all reached the hypopharyngeal region and formed hypoglossal muscle. Likewise, in *cMet* and *Lbx*1 mutants hypoglossal muscle develops (Bladt et al., 1995; Brohmann et al., 2000; Dietrich et al., 1999; Gross et al., 2000; Prunotto et al., 2004; Schäfer and Braun, 1999). Migratory muscle precursors are a synapomorphy (=newly acquired feature) of osteichthyans (Cole et al., 2011; Neyt et al., 2000), and in chondrichthyans and agnathans hypopharyngeral/hypoglossal muscle forms from somitic extensions (Kuratani et al., 1999; Kusakabe and Kuratani, 2005; Neal, 1897); reviewed in (Edgeworth, 1935; Goodrich, 1958). Similarly, non-migratory muscle precursors in the chicken and mouse all projected towards the circumpharyngeal path via lateral extensions (this study, (Bladt et al., 1995; Dietrich et al., 1999; Prunotto et al., 2004)). It thus appears that an overall stream of rostrally moving cells may be the evolutionarily basic force that underpins the transport of the originally non-migratory vertebrate HMP.

Neural crest cells have been shown to influence the differentiation of HMP, but neural crest or hypoglossal nerve ablations or genetic manipulation do not affect HMP deployment (Han et al., 2012; Hosokawa et al., 2010; Mackenzie et al., 1998). Moreover, our labelling experiments showed that the lateral mesoderm leads and envelopes the HMP as well as the neural crest cells. Thus, although the strict test of neural crest ablations combined with rendering HMP non-migratory has not yet been performed, it seems likely that the lateral mesoderm, possibly in cooperation with the overlying ectoderm may be the prime mover.

Interestingly, our labelling experiments revealed that the lateral mesoderm from the lower occipital and upper cervical area projects laterally–caudally. In the mouse, muscle precursors at the occipital–cervical interface are known to project ventrolaterally towards the septum transversum to form the diaphragm muscle (Dietrich et al., 1999); reviewed in (Merrell and Kardon, 2013). Thus, it is possible that also the caudal stream of lateral mesoderm cells may contribute to the coordination of overall cell movements. Yet, in *cMet*, and *Pax*3 mutants, while hypoharyngeal muscles form, diaphragm muscles are absent (Bladt et al., 1995; Dietrich et al., 1999; Franz, 1993; Franz et al., 1993; Maina et al., 1996; Prunotto et al., 2004; Tremblay et al., 1998; this study). Thus, the forces that work in the hypopharyngeal region may be unique.

### Towards deciphering the mechanism that controls the occipital morphogenetic movements

**Fig. 13:**
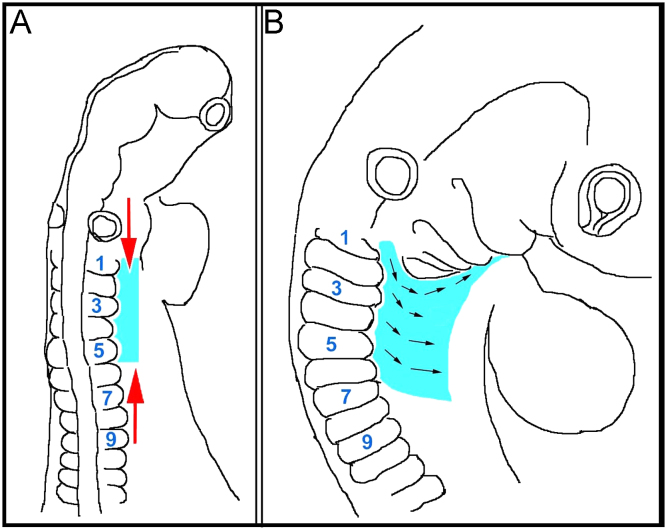
Summary of the morphogenetic movements of the lateral mesoderm and ectoderm at the head–trunk interface, projected onto the avian embryo. During the growth of the chick embryo between HH10 (E1$\frac{1}{2}$) and HH20 (E3), the trunk lateral mesoderm and ectoderm becomes rostrocaudally compressed (A, red arrows) but mediolaterally expanded (B, black arrows). Eventually, tissues originating from a position next to the most rostral somites move around the pharyngeal arches and along the floor of the pharynx towards the mandibular arch. The morphogenetic movements may be driven by relative growth processes, by space constraints executed by increasing cranial flexure and the swelling pharyngeal arches, by convergence extension movements or by concerted active migration of lateral mesodermal and ectodermal cells. Importantly, this cell movement begins before the release of neural crest cells and HMP, and serves as a conveyor belt for non-migratory cells, aiding the assembly of the hypopharyngeal apparatus.

During the time that the occipital cell movements described here take place, the pharynx closes ventrally. Thus, the occipital cell movements may represent the late phase of ventrally directed cell movements. At the time the occipital cells populate the circumpharyngeal path, the pharyngeal arches swell and the cranial flexure is established. We observed that longitudinal strips of lateral mesoderm grafted occipitally always became compressed in rostrocaudal and expanded in mediolateral direction. This compression was observed even when trunk lateral mesoderm was implanted (Dietrich, unpublished observations). Thus, it is possible that the compression of occipital cells into condensed streams and the rostral displacement of cells along the pharynx are passive and evoked by space constraints. In line with this idea, it has been suggested that the cervical flexure enforces the convergence of the in- and outflow poles of the heart and cardiac looping (Manner et al., 1993). However, in anamniotes the cranial flexure is far less pronounced, producing less of a constraint. Moreover, the rostrally projecting lateral mesoderm expresses *EphA*4,^2^ known to regulate cell cohesion and migration (Hirano et al., 1998). It therefore will have to be investigated in the future if the movement of the occipital lateral mesoderm discovered here involves active mechanisms or results from a combination of active movements and space constraints (summarised in Fig. 13).

## Author contributions

SD contrived the study; CLC, LEA and SD designed it; all authors contributed to the experiments; SD wrote and CS, LEA, KRW, MT and SD revised the manuscript.

## Footnotes

2 When cells undertake collective cells movements, they are able to drag non-migratory cells along. A prime example is the migration of border cells in the Drosophila ovary, see for example (Ramel et al., 2013).
