# Supplementary material for: Evolutionarily conserved morphogenetic movements at the vertebrate head–trunk interface coordinate the transport and assembly of hypopharyngeal structures
Source: Dev Biol. 2014 Jun 15;390(2):231–46. doi: 10.1016/j.ydbio.2014.03.003 (PMC4010675; doi:10.1016/j.ydbio.2014.03.003)
Supplement: Supplementary file 1 — Supplementary Material [file mmc1.pdf]

Suppl. Material 1. Source of protocols, in situ probes and antibodies

	Chicken	Mouse	Xenopus	Zebrafish
**Protocol for WISH, double WISH**	(Alvares et al., 2003; Dietrich et al., 1998; Dietrich et al., 1997; Lours and Dietrich, 2005; Mootoosamy and Dietrich, 2002)	(Dietrich et al., 1999)	(Baker et al., 1995; Harland, 1991)	(Thisse and Thisse, 2008)
**Protocol for WISH followed by antibody staining or vibratome sectioning**				
**Protocol for antibody staining on cryo-sections**		(Hutcheson et al., 2009)		
**WISH probes**				
**Alx4**	(Takahashi et al., 1998)		Amplified using Xtalx4F 5’-ATGAATGCTGATCCCTGTGTGT-3’ and Xtalx4R 5’-CACCTGGGCATAGTTCTCCG-3’	Amplified using zfalx4bF 5’-CCATTCCATCAGGATTGTCC-3’ and zfalx4bR 5’-AAGCGTTCTCGTTTTCTCCA-3’
**Dlx2**	(Blentic et al., 2008)			
**Lbx1**	(Dietrich et al., 1998; Dietrich et al., 1997)	(Jagla et al., 1995; Dietrich et al., 1999)	Amplified using Xt/Xllbx1F 5’-ATGACTTCCAAAGATGAAGCC-3’ and Xt/Xllbx1R 5’-AATCGTTTCTCCAACTCGTA-3’	
**Lbx2**				Amplified using zflbx2F 5’-GTGACGCCTGAAGCATGGTGC-3’ and zflbx2R 5’-ATCTGGACGTCAGAATGTAGAGG-3’
**MyoD**	(Bober et al., 1994)			
**Paraxis**	(Šošic et al., 1997)			
**Pax1**	(Ebensperger et al., 1995)			
**Pax3**	(Goulding et al., 1993)			
**Prrx1**	Amplified using GgPrrx1F 5’-GCTATGCCCACGCCATGGAG-3’ and Prrx1R 5’-CCTTCTGCGAAGCAGCTGCC-3’	Amplified using MmPrrx1F 5’-CACGTTCTGGAGCGGCAACC-3’ and MmPrrx1R 5’-CCCAGGAGAGATAATCGGTTGG-3’		
**Sox 10**	(Blentic et al., 2008)			
**Wnt6**	(Schubert et al., 2002)			
**antibodies**	QCPN (Developmental Studies Hybridoma Bank) detecting quail cells	IgG1 My32 (Sigma) detecting fast Myosin		
	RMO-270 antibody (Zymed) detecting intermediate neurofilaments	NOQ7.5 40 (Sigma) detecting slow Myosin		

References:

Alvares, L. E., Schubert, F. R., Thorpe, C., Mootoosamy, R. C., Cheng, L., Parkyn, G., Lumsden, A. and Dietrich, S. (2003). Intrinsic, Hox-dependent cues determine the fate of skeletal muscle precursors. *Dev Cell* **5**, 379-390.

Baker, C. V., Sharpe, C. R., Torpey, N. P., Heasman, J. and Wylie, C. C. (1995). A Xenopus c-kit-related receptor tyrosine kinase expressed in migrating stem cells of the lateral line system. *Mech Dev* **50**, 217-28.

Blentic, A., Tandon, P., Payton, S., Walshe, J., Carney, T., Kelsh, R. N., Mason, I. and Graham, A. (2008). The emergence of ectomesenchyme. *Dev Dyn* **237**, 592-601.

Bober, E., Brand-Saberi, B., Ebensperger, C., Wilting, J., Balling, R., Paterson, B. M., Arnold, H. H. and Christ, B. (1994). Initial steps of myogenesis in somites are independent of influence from axial structures. *Development* **120**, 3073-3082.

Dietrich, S., Abou-Rebyeh, F., Brohmann, H., Bladt, F., Sonnenberg-Riethmacher, E., Yamaai, T., Lumsden, A., Brand-Saberi, B. and Birchmeier, C. (1999). The role of SF/HGF and c-Met in the development of skeletal muscle. *Development* **126**, 1621-1629.

Dietrich, S., Schubert, F. R., Healy, C., Sharpe, P. T. and Lumsden, A. (1998). Specification of the hypaxial musculature. *Development* **125**, 2235-2249.

Dietrich, S., Schubert, F. R. and Lumsden, A. (1997). Control of dorsoventral pattern in the chick paraxial mesoderm. *Development* **124**, 3895-3908.

Ebensperger, C., Wilting, J., Brand-Saberi, B., Mizutani, Y., Christ, B., Balling, R. and Koseki, H. (1995). Pax-1, a regulator of sclerotome development is induced by notochord and floor plate signals in avian embryos. *Anat. Embryol.* **191**, 297-310.

Goulding, M. D., Lumsden, A. and Gruss, P. (1993). Signals from the notochord and floor plate regulate the region-specific expression of two Pax genes in the developing spinal cord. *Development* **117**, 1001-1016.

Harland, R. M. (1991). In situ hybridization: an improved whole-mount method for Xenopus embryos. *Methods Cell Biol* **36**, 685-95.

Hutcheson, D. A., Zhao, J., Merrell, A., Haldar, M. and Kardon, G. (2009). Embryonic and fetal limb myogenic cells are derived from developmentally distinct progenitors and have different requirements for beta-catenin. *Genes Dev* **23**, 997-1013.

Jagla, K., Dollé, P., Mattei, M.-G., Jagla, T., Schuhbaur, B., Dretzen, G., Bellard, F. and Bellard, M. (1995). Mouse Lbx1 and human LBX1 define a novel mammalian homeobox gene family related to the Drosophila ladybird genes. *Mech. Dev.* **53**, 345-356.

Lours, C. and Dietrich, S. (2005). The dissociation of the Fgf-feedback loop controls the limbless state of the neck. *Development* **132**, 5553-5564.

Mootoosamy, R. C. and Dietrich, S. (2002). Distinct regulatory cascades for head and trunk myogenesis. *Development* **129**, 573-583.

Schubert, F. R., Mootoosamy, R. C., Walters, E. H., Graham, A., Tumiotto, L., Munsterberg, A. E., Lumsden, A. and Dietrich, S. (2002). Wnt6 marks sites of epithelial transformations in the chick embryo. *Mech Dev* **114**, 143-148.

Šošic, D., Brand-Saberi, B., Schmidt, C., Christ, B. and Olson, E. N. (1997). Regulation of paraxis expression and somite formation by ectoderm- and neural tube-derived signals. *Dev Biol* **185**, 229-243.

Takahashi, M., Tamura, K., Buscher, D., Masuya, H., Yonei-Tamura, S., Matsumoto, K., Naitoh-Matsuo, M., Takeuchi, J., Ogura, K., Shiroishi, T. et al. (1998). The role of Alx-4 in the establishment of anteroposterior polarity during vertebrate limb development. *Development* **125**, 4417-4425.

Thisse, C. and Thisse, B. (2008). High-resolution in situ hybridization to whole-mount zebrafish embryos. *Nat Protoc* **3**, 59-69.

Suppl. Material 2. *In ovo* microsurgery

Tissue separation experiments

Tissue separation experiments were carried out in the occipital region of HH9-HH10 chicken embryos in ovo, implanting a 10µm thick piece of tantalum foil as impermeable barrier. To separate the occipital somites from the neural tube and notochord, a vertical incision was made along the wall of the neural tube, and the foil was pushed into the slit until it made contact with the endoderm (Suppl. Fig.2 C,D, Suppl. Fig.4a C,D). To separate the occipital somites from the lateral mesoderm, a vertical incision was made lateral to the somites, and the foil was inserted into the slit as before (Suppl. Fig.2 E,F, Suppl. Fig.4a E,F). To separate the somites from the overlying ectoderm, a shallow incision was made into the ectoderm lateral to the somite, a drop of dispase solution (Sigma) at 1mg/ml was applied using an aspirator, the ectoderm was peeled back, the slit was rinsed with PBS, and the foil was inserted to overlie the somites (Suppl. Fig.2 G,H, Suppl. Fig.4a G,H). The methodology is described in detail in (Dietrich et al., 1998; Dietrich et al., 1997). Embryos were cultured for 36 hours to reach HH18-19.

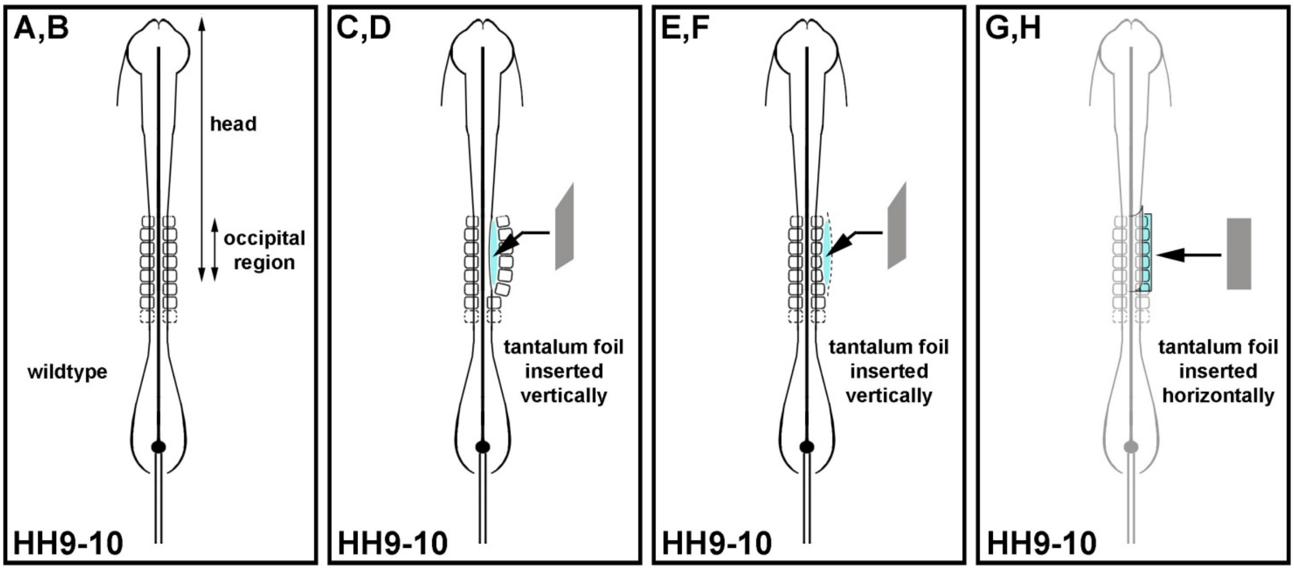

Suppl. Fig.2a. Separation of occipital somites from axial structures (C,D), lateral structures (E,F) and the overlying surface ectoderm (G,H).

Grafting experiments

(i) Implantation of rostrocaudally inverted occipital lateral mesoderm and ectoderm

Using flame-sharpened tungsten wire, the occipital lateral mesoderm and overlying ectoderm of the right side of HH9-10^{-} chicken embryos was excised *in ovo*. Stage-matched quail donors were pinned down in a Sylgard dish (Dow Corning), the left occipital lateral mesoderm and ectoderm were excised, aspired into a glass capillary to control its orientation, and released into the gap made in the host, dorsal (ectodermal) side up. This way, the rostrocaudal orientation of the graft was inverted while the original mediolateral orientation was maintained (Suppl.Fig.2b A,A'; Fig.9A). In a similar fashion, lateral mesoderm and ectoderm from cervical, forelimb or flank levels was grafted (not shown). Embryos were harvested 36 hours after surgery at HH18-19.

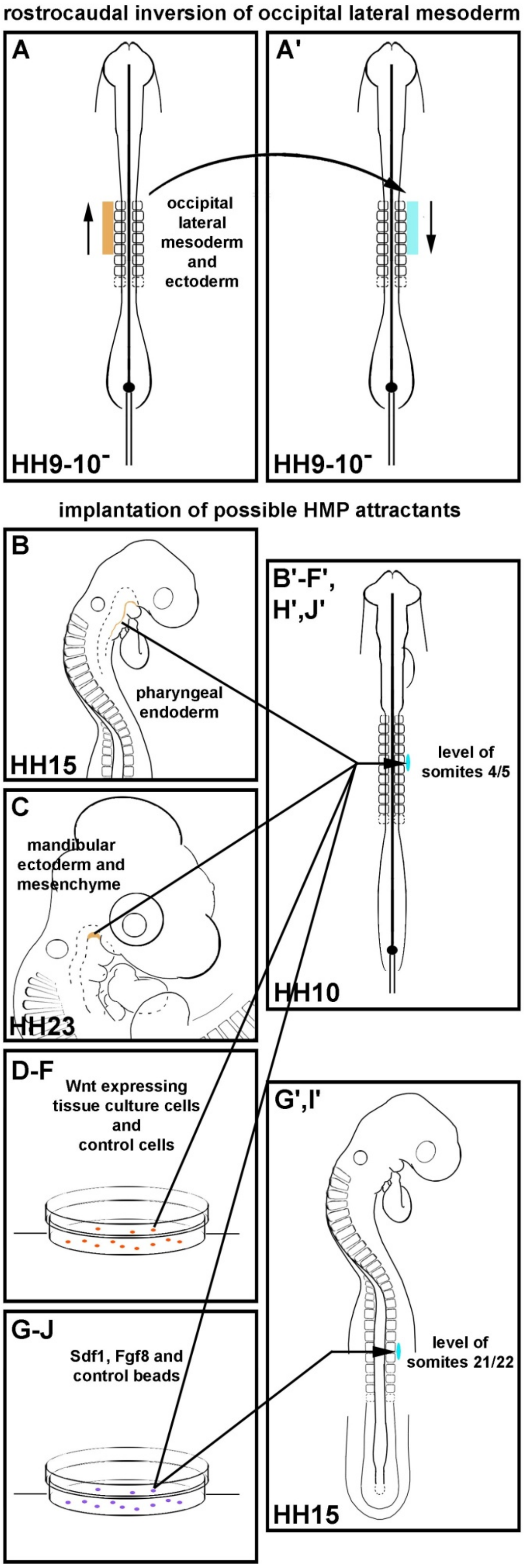

Suppl. Fig.2b. Grafting of possible HMP attractants. Quail derived grafts are shown in brown, CellTracker Orange stained tissue culture cells in orange, protein loaded beads in blue and recipient areas in the host in turquoise.

(ii) Implantation of putative HMP attractants next to the caudal-most occipital somite

Using flame-sharpened tungsten needles, an insertion was made next to somites 4/5 of a HH10 chicken embryo *in ovo*. Pharyngeal endoderm of a pinned-down HH15-16 quail embryo was separated from surrounding tissues by applying a drop of 1mg/ml dispase (Sigma) with an aspirator, the tissue was transferred into the host with a serum-coated tip and manoeuvred into the slit (Suppl. Fig.2b B,B'; Fig.9B). Oral ectoderm and mesenchyme was excised from pinned-down HH23 donors using tungsten needles, and transferred to the host using serum-coated tips (Suppl. Fig.2b C,C'; Fig.9C). Wnt expressing cells and control cells were stained with CellTracker Orange (Invitrogen)

and grafted as detailed in (Cheng et al., 2004) (Suppl. Fig.2b D-F,D'-F'; Fig.9D-F). Fgf8 (1mg/ml), Sdf1 (1mg/ml) or BSA (1mg/ml; control) loaded heparin-coated acrylic beads (Sigma) were prepared and grafted as described in (Alvares et al., 2003) (Suppl. Fig.2b H,J,H'J'; Fig.9H,J). As control, beads were grafted next to the somites at the forelimb-flank boundary at HH15 (Suppl. Fig.2b G,I,G'I'; Fig.9G,I). Embryos were harvested at HH18-19.

(iii) Somite replacements

Using 100µm flame-sharpened tungsten wire, the occipital somites of HH10 or limb-level somites of HH15 chicken hosts were excised *in ovo* and replaced by HH9-10$^-$ occipital somites (control 1; Suppl. Fig.2c A,D; Fig.10A), HH15 limb level somites (control 2, not shown), HH15 flank level somites (Suppl. Fig.2c B,E; Fig.10B) or HH8-9$^-$ pre-otic head mesoderm (Suppl. Fig.2c C,F; Fig.10C,F), with grafts derived from quail donors, as described by (Alvares et al., 2003; Mootoosamy and Dietrich, 2002). When the strips of explanted quail somites adhered well, larger strips were grafted (Fig.10D). To simultaneously explore the behaviour of cells exposed to a limb and non-limb- environment, somites were grafted to the fore limb-flank border (Fig.10E). Embryos were harvested at stage HH20.

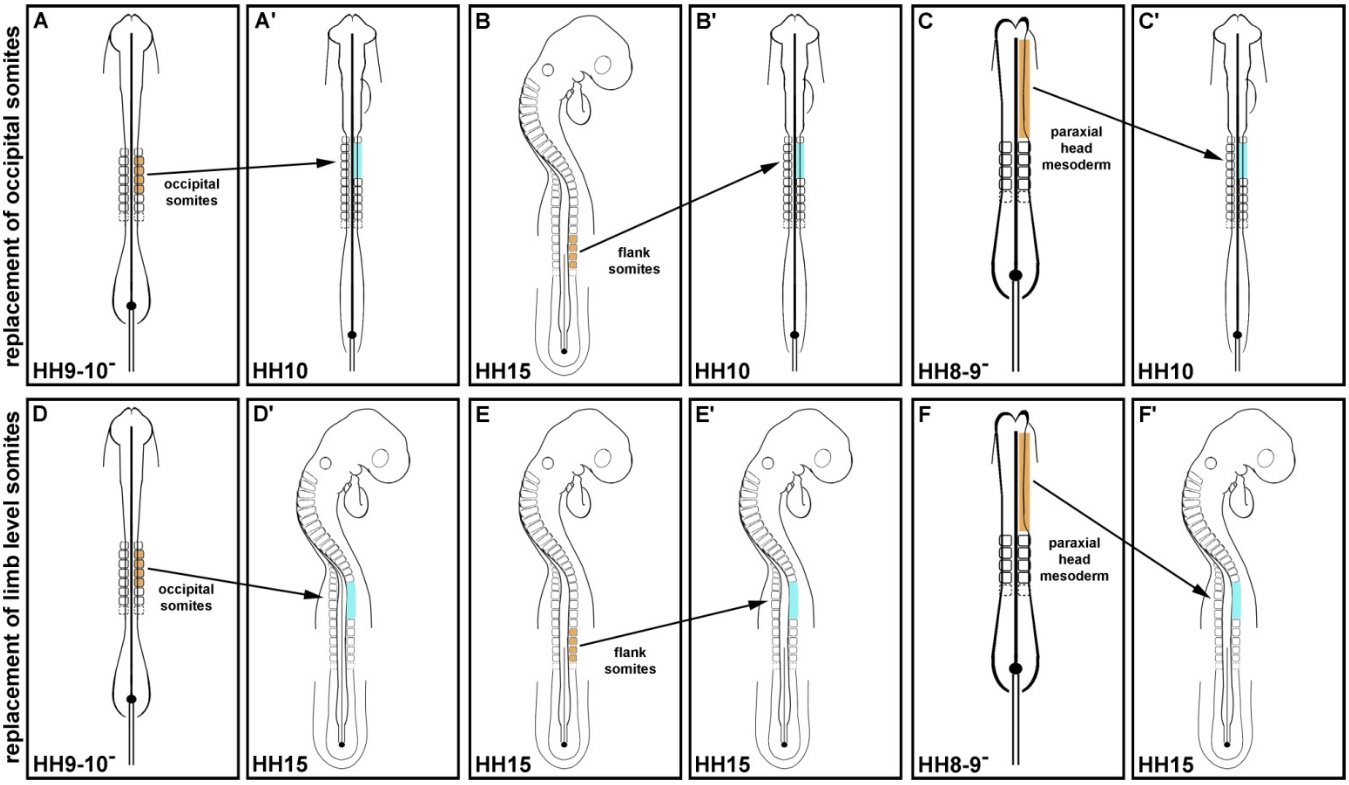

Suppl. Fig.2c. Somite replacement experiments. Quail derived tissues are shown in brown, recipient areas in the host in turquoise. Embryos not drawn to scale.

References:

Alvares, L. E., Schubert, F. R., Thorpe, C., Mootoosamy, R. C., Cheng, L., Parkyn, G., Lumsden, A. and Dietrich, S. (2003). Intrinsic, Hox-dependent cues determine the fate of skeletal muscle precursors. *Dev Cell* **5**, 379-390.

Cheng, L., Alvares, L. E., Ahmed, M. U., El-Hanfy, A. S. and Dietrich, S. (2004). The epaxial-hypaxial subdivision of the avian somite. *Dev Biol* **274**, 348-369.

Dietrich, S., Schubert, F. R., Healy, C., Sharpe, P. T. and Lumsden, A. (1998). Specification of the hypaxial musculature. *Development* **125**, 2235-2249.

Dietrich, S., Schubert, F. R. and Lumsden, A. (1997). Control of dorsoventral pattern in the chick paraxial mesoderm. *Development* **124**, 3895-3908.

Mootoosamy, R. C. and Dietrich, S. (2002). Distinct regulatory cascades for head and trunk myogenesis. *Development* **129**, 573-583.

Suppl. Material 3. Rostral extension of occipital gene expression domains is conserved in osteichthyans.

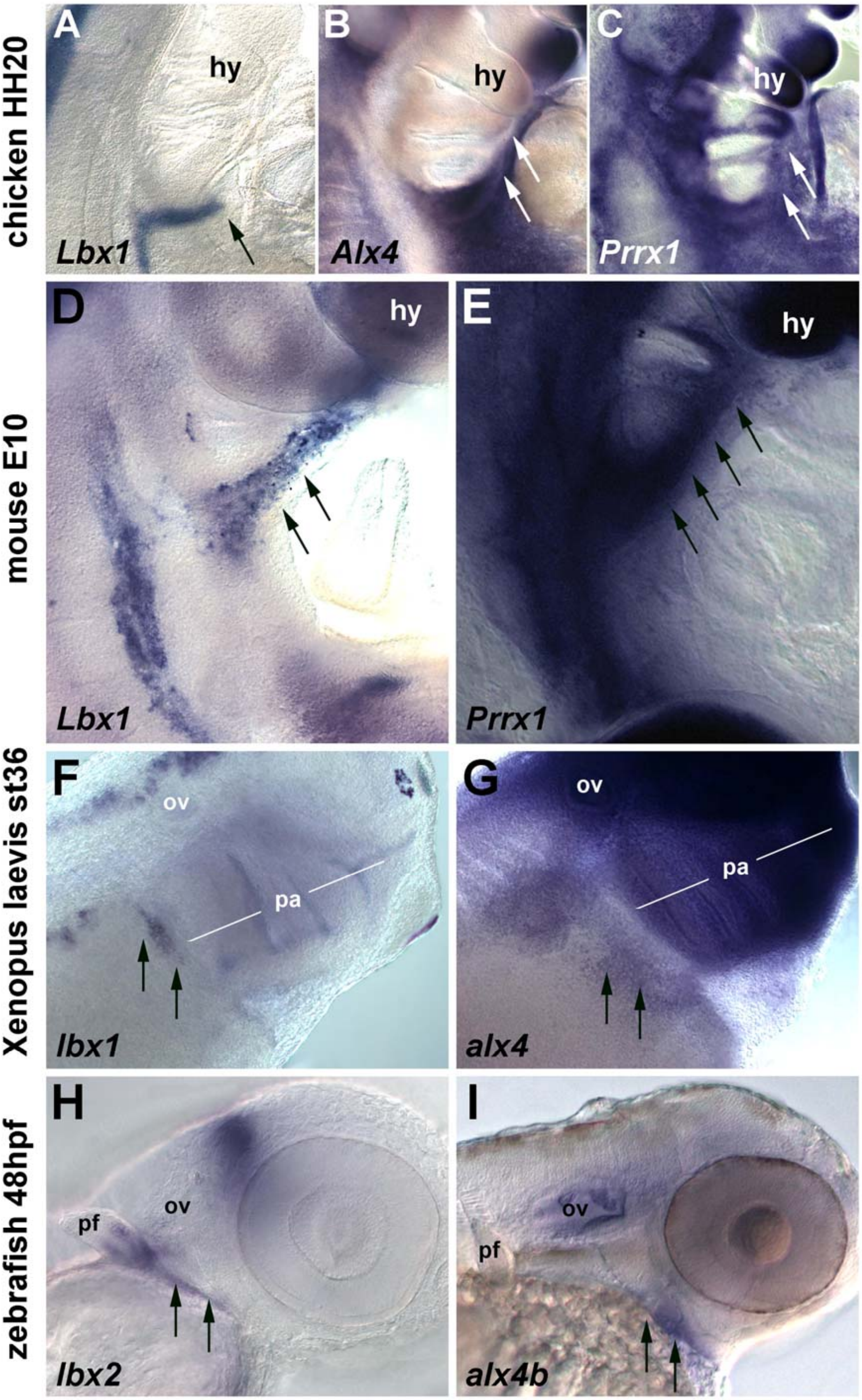

Head-trunk interface of (A-C) chicken embryos at HH20, (D,E) mouse embryos at E10.5 pc, (F,G) *Xenopus laevis* embryos at st36 and (H,I) zebrafish embryos at 48 hpf; lateral views, rostral to the top (A-E) or the right (F-I); the molecular markers used are indicated. The hypobranchial/hypoglossal muscle precursors (HMP) express *Lbx1* (A,D,F, arrows) or in the zebrafish, the paralogous gene *lbx2* (H, arrows). The paired type homeobox genes *Alx4* and *Prrx1* are expressed in the lateral mesoderm, with expression domains anticipating the path of the HMP (B,C,E,G,I arrows).

Abbreviations: hy, hyoid arch; ov, otic vesicle; pa, pharyngeal arches; pf, pectoral fin.

Chicken, mouse and Xenopus represent the sarcopterygian and zebrafish the actinopterygian class of osteichthyans ("bony" jawed vertebrates). The conserved expression patterns shown here suggest a shared developmental mechanism that was present deep in osteichthyan evolution.

In chicken, mouse and Xenopus, *Lbx1* labelled the emigrating HMP on their circumpharyngeal path (Suppl. Fig.3A,D,F; arrows). In the zebrafish, while the closely related *lbx1a, 1b* and *2* genes marked migratory muscle precursors for the pectoral fins (Neyt et al., 2000; Ochi and Westerfield, 2009; Thisse et al., 2004; Wotton et al., 2008) and Wotton and Dietrich, unpublished observations), *lbx2* was expressed in HMP (Suppl. Fig.3H, arrows). In chicken, mouse and zebrafish, *Alx4* (zebrafish: *alx4b*) labelled the lateral mesoderm surrounding the most caudal pharyngeal arch and the hypopharyngeal region (Suppl. Fig.3B,G,I; arrows), in the frog accompanied by expression in neural crest cells filling the arches (Suppl. Fig.3G, pa). In the mouse, circumpharyngeal expression of *Alx4* was low, but in both chicken and mouse, *Prrx1* showed the same, rostrally expanding expression along the floor of the pharynx (Suppl. Fig.3C,E; arrows). Notably, in all species, the lateral mesoderm markers spread into hypopharyngeal areas before the HMP.

Suppl. Material 4. Occipital somite patterning and HMP specification.

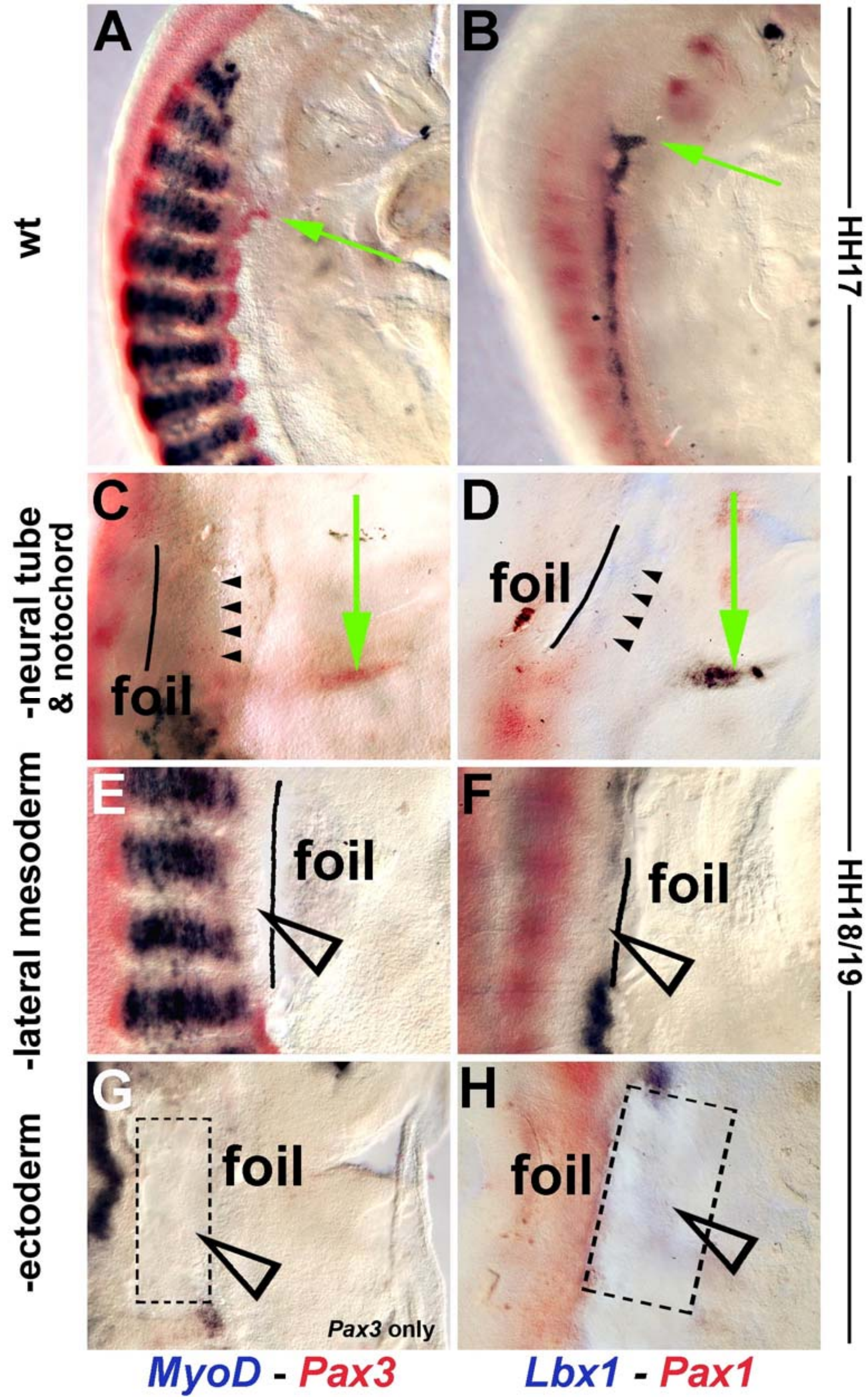

Lateral views of the occipital region of chicken embryos at HH17 (A,B) and 36 hours after operation at HH18-19 (C-H), rostral to the top, lateral to the right. (A,C,E) are stained for the expression of *Pax3* (dermomyotome and migratory muscle precursors; red) and *MyoD* (epaxial myotome; blue), (B,D,F,H) for *Lbx1* (migratory muscle precursors; blue) and *Pax1* (sclerotome; red); (G) is stained for *Pax3* only (blue). HMP express *Pax3* and *Lbx1* before (not shown) and during deepithelialisation from the occipital somites (A,B; arrows) and on route to the floor of the mandibular arch (C,D; arrows). (C,D) Separation of the occipital somites from neural tube and notochord using tantalum foil eliminates expression of *MyoD* (C, arrowheads) and *Pax1* (D, arrowheads), but leaves expression of *Pax3* (C, arrow) and *Lbx1* (D, arrow) undisturbed. Separation of the occipital somites from the lateral mesoderm (E,F) or the surface ectoderm (G,H) leaves *MyoD* and *Pax1* expression (E,F,H) intact but prevents expression of *Pax3* (E,G, open arrowheads) and *Lbx1* (F,H, open arrowheads). Thus, the patterning of the occipital somites and the formation of HMP mirrors the patterning of limb somites and the formation of limb muscle precursors (Dietrich et al., 1998). Abbreviations: wt, wildtype.

Suppl. Material 5. Model for the evolution of developmental mechanisms underpinning the assembly of the hypopharyngeal apparatus in vertebrates.

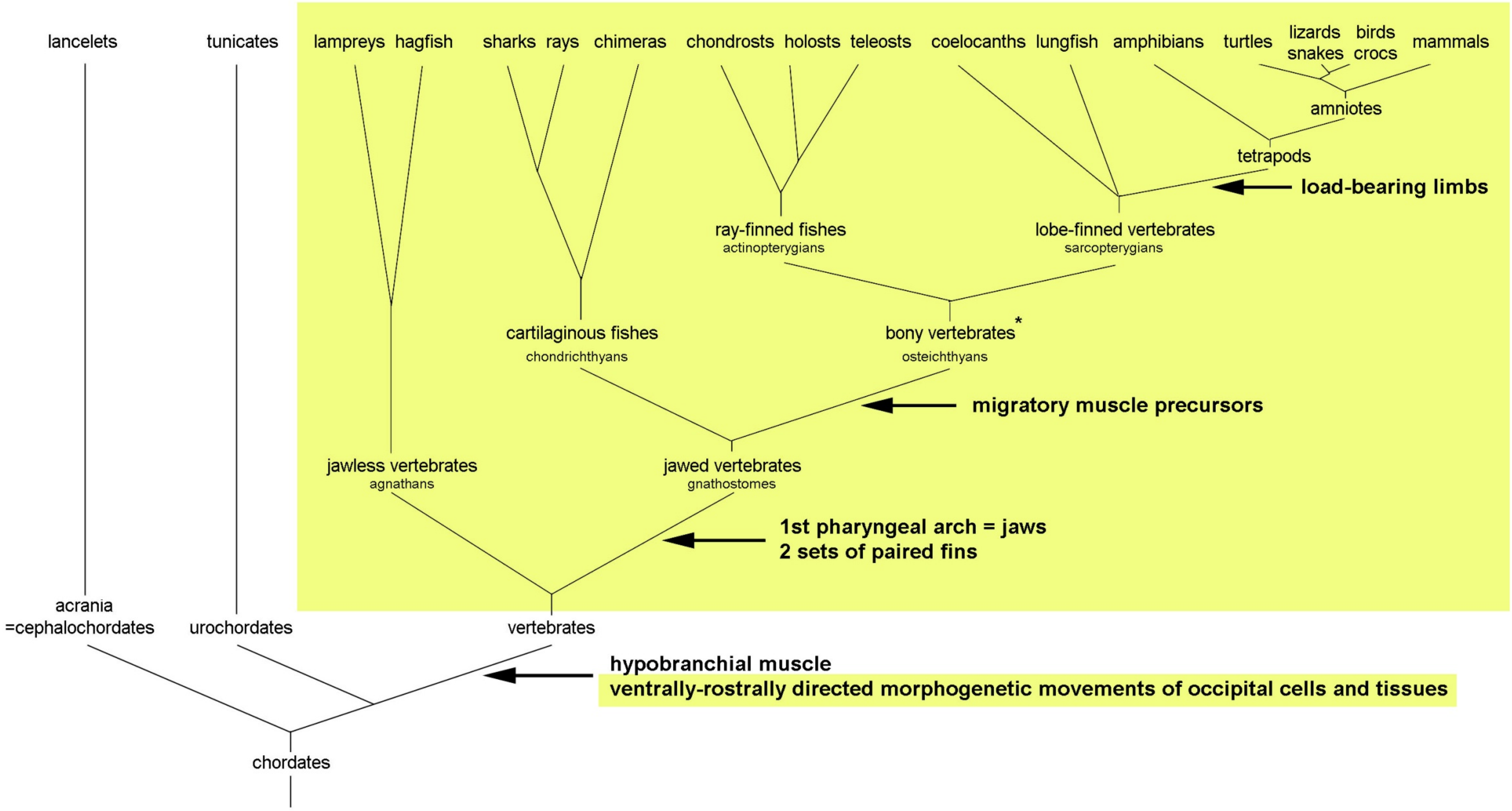

Chordate evolutionary tree (crown groups only). Deep in the evolution of vertebrates, a mechanism driven by cell movements of the occipital lateral mesoderm and ectoderm evolved that allowed the transport of occipital derivatives along the pharynx. In the lineage leading to osteichthyans, this mechanism was superseded when migratory muscle precursors evolved. However, when occipital migratory muscle precursors are rendered non-migratory, the evolutionarily ancient mechanisms can be visualised as muscle precursors are able to their target site regardless.

*Osteichthyans are commonly known as bony vertebrates, but a recent study (Zhu et al., 2013) suggested that the ability to generate mineralised tissues evolved before the chondrichthyan-osteichthyan split and was secondarily lost in chondrichthyans.

References:

Zhu, M., Yu, X., Ahlberg, P. E., Choo, B., Lu, J., Qiao, T., Qu, Q., Zhao, W., Jia, L., Blom, H. et al. (2013). A Silurian placoderm with osteichthyan-like marginal jaw bones. *Nature* **502**, 188-93.
